# Cell-Based Screen Identifies Human Interferon-Stimulated Regulators of *Listeria monocytogenes* Infection

**DOI:** 10.1371/journal.ppat.1006102

**Published:** 2016-12-21

**Authors:** Sofya S. Perelman, Michael E. Abrams, Jennifer L. Eitson, Didi Chen, Alyssa Jimenez, Marcel Mettlen, John W. Schoggins, Neal M. Alto

**Affiliations:** 1 Department of Microbiology, University of Texas Southwestern Medical Center, Dallas, Texas, United States of America; 2 Department of Cell Biology, University of Texas Southwestern Medical Center, Dallas, Texas, United States of America; McMaster University, CANADA

## Abstract

The type I interferon (IFN) activated transcriptional response is a critical antiviral defense mechanism, yet its role in bacterial pathogenesis remains less well characterized. Using an intracellular pathogen *Listeria monocytogenes* (*Lm*) as a model bacterial pathogen, we sought to identify the roles of individual interferon-stimulated genes (ISGs) in context of bacterial infection. Previously, IFN has been implicated in both restricting and promoting *Lm* growth and immune stimulatory functions *in vivo*. Here we adapted a gain-of-function flow cytometry based approach to screen a library of more than 350 human ISGs for inhibitors and enhancers of *Lm* infection. We identify 6 genes, including *UNC93B1*, *MYD88*, *AQP9*, and *TRIM14* that potently inhibit *Lm* infection. These inhibitors act through both transcription-mediated (MYD88) and non-transcriptional mechanisms (TRIM14). Further, we identify and characterize the human high affinity immunoglobulin receptor FcγRIa as an enhancer of *Lm* internalization. Our results reveal that FcγRIa promotes *Lm* uptake in the absence of known host *Lm* internalization receptors (E-cadherin and c-Met) as well as bacterial surface internalins (InlA and InlB). Additionally, FcγRIa-mediated uptake occurs independently of *Lm* opsonization or canonical FcγRIa signaling. Finally, we established the contribution of FcγRIa to *Lm* infection in phagocytic cells, thus potentially linking the IFN response to a novel bacterial uptake pathway. Together, these studies provide an experimental and conceptual basis for deciphering the role of IFN in bacterial defense and virulence at single-gene resolution.

## Author Summary

While the type I interferon response is known to be activated by both viruses and bacteria, it has mostly been characterized in terms of its antiviral properties. *Listeria monocytogenes*, an opportunistic Gram-positive bacterial pathogen with up to 50% mortality rate and a variety of clinical manifestations, is a potent activator of interferon secretion. In mouse models, interferon has been previously implicated in both restricting and promoting *L*. *monocytogenes* infection. Here, we utilized a high-throughput flow-cytometry based approach to screen a library of human interferon I stimulated genes (ISGs) and identified regulators of *L*. *monocytogenes* infection. These include inhibitors that act through both transcriptional (MYD88) and transcription-independent (TRIM14) mechanisms. Strikingly, expression of the human high affinity immunoglobulin receptor FcγRIa (CD64) was found to potently enhance *L*. *monocytogenes* infection. Both biochemical and cellular studies indicate that FcγRIa increases primary invasion of *L*. *monocytogenes* through a previously uncharacterized IgG-independent internalization mechanism. Together, these studies provide an important insight into the complex role of interferon response in bacterial virulence and host defense.

## Introduction

Mammalian cells encode numerous pattern recognition receptors (PRRs) that sense invading pathogens and initiate innate immune responses through cytokine and chemokine production [1]. With viral pathogens, the type I interferon (IFN) family of cytokines serves as a first line of defense and is essential for controlling virus replication and pathogenesis. The IFN-induced antiviral response results from the transcription of hundreds of interferon-stimulated genes (ISGs), many of which inhibit different steps of the viral life cycle [2, 3]. Although less studied, the type I IFN response is also induced by many bacterial pathogens including *Legionella pneumophila*, *Helicobacter pylori*, *Francisella tularensis*, *Yersinia pseudotuberculosis*, *Mycobacterium tuberculosis*, and *Listeria monocytogenes* [4]. However, the role of type I IFN in bacterial infection remains unclear and systematic studies to uncover the breadth of ISGs targeting a bacterial pathogen have not been carried out.

We chose to clarify these aspects of IFN biology by using *Listeria monocytogenes* (herein referred to as *Lm*) as a model pathogen as its cellular life cycle has been described in detail and it exhibits a complex relationship with the mammalian IFN response system [5]. *Lm* is a Gram-positive food-borne pathogen that causes severe and life threatening disease in immunocompromised individuals, pregnant women, elderly and children [6]. Upon invasion of enterocytes, hepatocytes, or phagocytes, *Lm* gains access to the cytoplasm by lysing the primary phagosome. *Lm* rapidly replicates in the cytoplasm and spreads to adjacent cells via actin-based protrusion machinery [7]. Recent studies show that *Lm* stimulates the type I IFN response by secreting cyclic diadenosine monophosphate (c-di-AMP) that activates the Stimulator of Interferon Genes (STING). Activation of STING results in IRF3 phosphorylation and transcription of IFN genes [8, 9]. Notably, STING-deficient mice fail to produce IFNβ in response to *Lm* infection [10].

While the relationship between IFN and *in vivo Lm* infection has been firmly established, some discrepancies do exist between these studies. Early work showed that IFNβ increases the tolerance of mice to intravenous systemic *Lm* infection [11]. Similarly, *Ifnar1* is required for resistance of mice to *Lm* invasion through the intestinal tract, further demonstrating a protective effect of IFN for a natural route of infection [12]. However, more recent studies indicate that mice lacking a functional type I IFN receptor (*Ifnar1*-/-) display greater resistance to intravenous *Lm* infection, suggesting that IFN exacerbates systemic *Lm* infection [13–15]. The type I IFN response has also been found to suppress adaptive immunity against *Lm*, since *Sting*-deficient mice exhibit greater numbers of cytotoxic lymphocytes and show protection from *Lm* reinfection after immunization [16]. These various effects of type I IFN on *Lm* infection likely reflect the different routes of *Lm* infection and the pleiotropic roles of IFN in distinct tissue environments or cellular populations encountered by the pathogen. Nevertheless, it is clear that type I IFN plays a significant role in shaping the host-pathogen interaction *in vivo*.

Because IFN induces a robust transcriptional response, the regulatory role of IFN in bacterial infection likely depends on the cellular expression of ISGs. However, the functions of most ISGs in immunity have not yet been elucidated due to the technical challenges of studying complex transcriptional responses at single-gene resolution. Recently, overexpression screens have been designed to study individual ISG functions [17–20]. While these approaches have proven to be highly successful for identifying genes that potently suppress invasion, replication, or egress of a wide variety of viruses, similar screening methodologies have not yet been adapted for bacterial pathogens. Here, we performed a gain-of-function screen of over 350 human type I IFN ISGs to identify genes that regulate *Lm* infection. This screen revealed potent bacterial restriction factors including MYD88, UNC93B1, TRIM14, AQP9, and MAP3K14. We demonstrated that the signaling adaptor MYD88 restricts *Lm* infection through the stimulation of a robust host gene expression program. In contrast, TRIM14 inhibited *Lm* infection through a non-transcriptional mechanism, thus suggesting that IFN stimulates diverse antibacterial properties. Importantly, we identified the human high affinity immunoglobulin receptor FcγRIa (CD64) as an IgG-independent enhancer of *Lm* internalization and established its role in the entry of *Lm* into phagocytic cells. Taken together, these findings reveal effector molecules involved in the complex relationship between *Lm* and the IFN response system, and open new avenues for exploring the cell-autonomous immune regulation of other bacterial pathogens.

## Results

### Fluorescence-based gain-of-function screening approach

**Fig 1:**
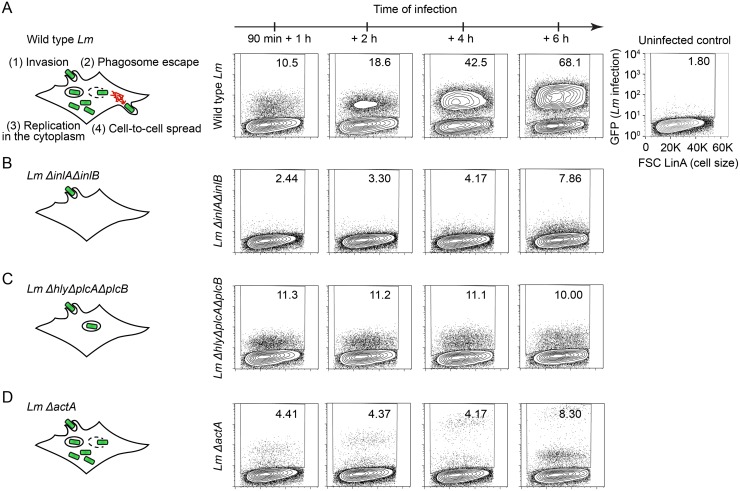
Fluorescence-based screening approach. (A-D) Diagram illustrating cellular lifecycle of wild type *Lm* (A), *Lm ΔinlAΔinlB* (B), *Lm ΔhlyΔplcAΔplcB* (C), *Lm ΔactA* (D). Representative flow cytometry plots of *STAT1*-deficient fibroblasts infected with GFP-expressing wild type *Lm* (A), *Lm ΔinlAΔinlB* (B), *Lm Δhly ΔplcA ΔplcB* (C), and *Lm ΔactA* (D) strains for 1, 2, 4, and 6 h following 1.5 h initial infection. Values in the upper right corner of each plot indicate the percentage of GFP-positive cells in singlet cell population. The uninfected control is presented on the right.

We sought to employ a gain-of-function screening approach to identify ISGs that regulate *Lm* infection of host cells. We first optimized the screening conditions by determining the suitability of the host cell type previously used for ISG screens [17, 18] and by defining the optimal conditions of *Lm* infection. Since *Lm* is known to potently induce IFN expression [5], we chose human *STAT1*-deficient fibroblasts as the primary host cell type for infection [21]. These cells lack functional *STAT1*, have defective IFN responses, and therefore limit the spurious activation of ISGs during bacterial infection. To screen hundreds of ISGs in a single experiment, we optimized *Lm* infection of *STAT1*-deficient fibroblasts for compatibility with multicolor flow cytometry with auto-sampling functionality [17]. GFP-expressing *Listeria monocytogenes* 10403s (GFP*-Lm*) was added to fibroblasts at a multiplicity of infection (MOI) 5 in the absence of antibiotics. GFP-*Lm* was incubated for 90 minutes with host cells prior to adding gentamicin-containing media to eliminate non-internalized extracellular bacteria. Approximately 10% of host cells were infected by GFP-*Lm* at this time point. Cells were then incubated for 1, 2, 4 and 6 hours, providing a temporal evaluation of infection. As shown in Fig 1A, we observed an increase in the percentage of GFP-positive host cells over time indicating that GFP-*Lm* readily infects *STAT1*-deficient fibroblasts.

*Lm* infection progresses through a series of well-defined stages including entry, vacuole escape, cytoplasmic replication, and cell-to-cell spread (Fig 1A). Since ISGs could potentially affect any stage of the *Lm* lifecycle, we assessed the ability of flow cytometry to identify blocks in each of these distinct stages. *STAT1*-deficient fibroblasts were infected with mutant *Lm* strains that lack key virulence factors that are critical for cellular entry (*Lm ΔinlAΔinlB*), phagosomal escape *(Lm ΔhlyΔplcAΔplcB)*, and cell-to-cell spread *(Lm ΔactA*). As expected, *Lm ΔinlAΔinlB*, lacking the major *Lm* invasion proteins InlA and InlB, exhibited a severe infection defect observed as early as 1 hour following initial infection (Fig 1B). In contrast, *Lm ΔhlyΔplcAΔplcB* mutant lacking listeriolysin O (LLO) and phospholipases required for phagosomal rupture and escape, invaded cells similar to wild type *Lm*, yet the percentage of infected cells and their bacterial burden (intensity of the GFP signal) did not increase over the time course of infection (Fig 1C). This observation is consistent with the *Lm ΔhlyΔplcAΔplcB* phenotype in which the pathogen trapped in the phagosome survives but is unable to replicate [22–25]. Finally, *Lm ΔactA* lacking the ActA protein required for intracellular actin-based motility invaded cells initially but failed to spread from cell to cell. Importantly, the GFP fluorescence intensity of infected *STAT1-*deficient fibroblasts increased over the course of infection due to bacterial replication and accumulation in initially invaded cells (Fig 1D). Thus, flow cytometry is a well-suited method to measure *Lm* infection of *STAT1*-deficient fibroblasts as it is capable of detecting specific infection defects that may arise due to ISG expression.

### Type I ISG screen identifies potent regulators of *Lm* infection

**Fig 2:**
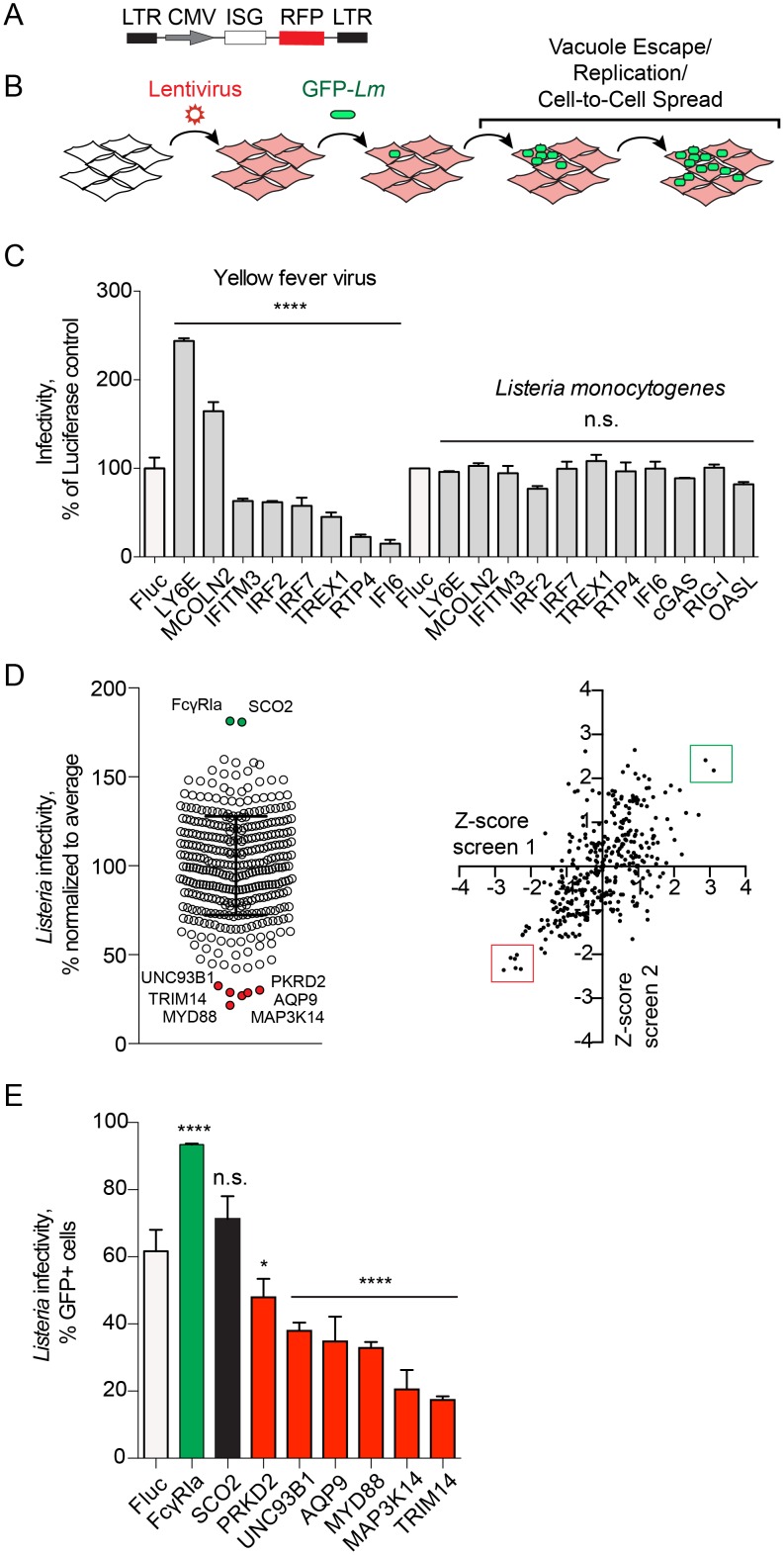
Flow cytometry-based gain-of-function screen identifies regulators of *Lm* infection. (A) Schematic of the bicistronic lentiviral vector; CMV, immediate early promoter from human cytomegalovirus; LTR, HIV-1 long terminal repeat. (B) Diagram illustrating the gain-of-function fluorescence-based screen for regulators of *Lm* infection. (C) YFV and *Lm* infectivity in the presence of ISG inhibitors and enhancers of viral infection. Infectivity was measured by flow cytometry as a percentage of GFP-positive cells in RFP-positive population and normalized to a Fluc control for each pathogen (white bars). Error bars represent s.d., n = 3 (YFV), n = 2 (*Lm*). Statistical significance was determined by one-way analysis of variance (ANOVA) for each pathogen prior to normalization (****, P<0.0001; n.s., not significant). (D) (*Left*) Dot plot of *Lm* infectivity in the presence of expressed ISGs. *Lm* infectivity was measured by flow cytometry in two replicate screens and presented as an average. Error bars represent s.d., n = 2. (*Right*) Scatter plot of Z-scores of screen replicates 1 and 2. Genes selected for further confirmation are labeled (*left*) and boxed (*right*). (E) Infectivity of *Lm* in *STAT1*-deficient fibroblasts transduced with lentivirus expressing Fluc (white bar) and selected ISGs from the large-scale screen in (D). *Lm* infectivity was measured similarly to Fig 2C. Error bars represent s.d., n = 3 (*, P<0.05; ****, P<0.0001; n.s., not significant).

We next asked whether ISGs that control viral infection also regulate bacteria. Briefly, *STAT1*-deficient fibroblasts were transduced with bicistronic lentiviral vectors driving constitutive expression of an ISG and a red fluorescent protein TagRFP (Fig 2A). Cells expressing ISGs in a one-gene one-well format were then challenged with a GFP-*Lm* and resulting infection was analyzed by flow cytometry (Fig 2B). Infection rates were quantified as a percentage of GFP-positive host cells (a measure of infection) among the RFP-positive cell population (a measure of ISG expression). Firefly luciferase (Fluc), which did not affect *Lm* infection (S1 Fig), was used as a negative control. A panel of ISGs that enhance (*MCOLN2*, *LY6E*) or inhibit (*IFI6*, *RTP4*, *TREX1*, *IRF2*, *IRF7*, *P2RY6*, and *IFITM3*) yellow fever virus (YFV) had no effect on *Lm* infection (Fig 2C). In addition, in the absence of IFN signaling the cytosolic DNA and RNA sensors *MB21D1* (cGAS) and *DDX58* (RIG-I) [26, 27] as well as *OASL*, an ISG that inhibits hepatitis C virus [17, 28] did not inhibit *Lm* (Fig 2C). Thus, effects of ISGs can be differentiated between model bacterial and viral pathogens.

Next, we expressed a library of over 350 ISGs in a one-gene to one-well format [17] and performed *Lm* infection as described above. Infectivity of *Lm* obtained from the average of two screen replicates is shown as a dot plot in Fig 2D (S1 Table). The majority of ISGs had little effect on *Lm* infection with the cellular bacterial burdens falling within two standard deviations of the population mean (Z-score less than 2, Fig 2D). We considered inhibitors of *Lm* infection as those ISGs that restricted infection with Z-score greater than 2. Six ISGs fulfilled these criteria including *PKRD2*, *UNC93B1*, *MYD88*, *AQP9*, *MAP3K14*, and *TRIM14* (Fig 2D). In addition, two genes *FCGR1A* and *SCO2* enhanced *Lm* infection with Z-score greater than 2. Repeat trials with independent lentiviral preparations confirmed statistically significant inhibitory effects for all six ISGs and an enhancing effect for *FCGR1A* (Fig 2E).

### Toll-like receptor signaling components identified as inhibitors of *Lm* infection

**Fig 3:**
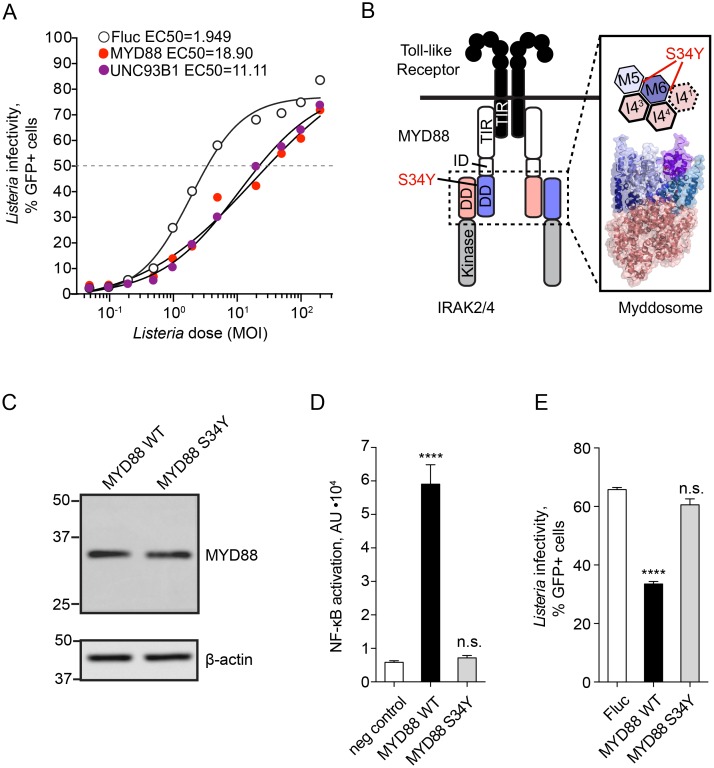
MYD88 induces an anti-bacterial transcriptional response. (A) Infectivity of *Lm* in *STAT1*-deficient fibroblasts transduced with lentivirus expressing Fluc (empty circles), *MYD88* (red) and *UNC93B1* (purple) was tested over a range of MOI. Dose-response curves were fitted to a four-parameter sigmoidal model and EC50 values calculated using GraphPad Prism software: EC50_Fluc_ = 1.949, EC50_MYD88_ = 18.90, EC50_UNC93B1_ = 11.11. (B) Diagram illustrating Toll-like receptor (TLR) signaling complex (left) and fragment of planar arrangement of the Myddosome complex (PDB 3MOP) and localization of the MYD88 Ser34 between MYD88 M5 and M6 as well as MYD88 M6 and IRAK I4^1^ is shown. (C) Western blot analysis of MYD88 expression in *STAT1*-deficient fibroblasts transduced with lentivirus expressing wild type and S34Y mutant MYD88. Equal amounts of each lysate (30μg total protein as measured by BCA assay) were loaded per lane. Actin is shown below as a loading control. (D) NF-κB-luciferase activity in untransduced *STAT1*-deficient fibroblasts (negative control, white bar), transduced with lentivirus expressing wild type (WT, black bar), or S34Y mutant MYD88 (S34Y, grey bar) and transfected with the reporter plasmid pNF-κB-luciferase. Error bars represent s.d., n = 3. Statistical significance was determined by one-way analysis of variance (ANOVA) (****, P< 0.0001; n.s., not significant). (E) Infectivity of *Lm* in *STAT1*-deficient fibroblasts transduced with lentivirus expressing Fluc (white bar), wild type MYD88 (WT, black bar), or MYD88 S34Y mutant (S34Y, grey bar). *Lm* infectivity was measured by flow cytometry and statistical significance determined by one-way ANOVA, error bars represent s.d., n = 3 (****, P< 0.0001, n.s., not significant).

The identification of *UNC93B1* and *MYD88* as cell-intrinsic inhibitors of *Lm* provided positive validation of the ISG screen. These genes are key components of the immune response to pathogens and function to properly target Toll-like Receptors (TLR) to subcellular compartments and to propagate NF-κB signal transduction, respectively [29, 30]. Consistent with the initial ISG screen, a dose-response to infection revealed that *MYD88* and *UNC93B1* reduced *Lm* infectivity (defined as the MOI of *Lm* required to achieve 50% cellular infection during the course of 8 hours) by 9.7-fold and 5.7-fold compared to firefly luciferase control (Fig 3A).

To determine if the anti-*Lm* activity of MYD88 results from increased NF-κB transcriptional response as predicted, we isolated mRNA from *STAT1*-deficient fibroblasts ectopically expressing MYD88. RNA-seq revealed 107 genes that were upregulated over 2-fold (S2 Table) and included NF-κB signature genes involved in inflammation (e.g. *IL6*, *IL8*, *IL1B*, *CXCL1-3*, *CXCL5*, *CCL2*), signal transduction (e.g. *NFKB1/2*, *RELB*, *IRAK2*, *C1QTNF1*), cell adhesion (e.g. *ICAM-1*, *LAMB3*, *MMPs*), and complement activation (C3) (S2 Table). To then establish the role of NF-κB activation in the observed antibacterial activity of MYD88, we tested the function of a naturally occurring single nucleotide polymorphism of *MYD88* (rs1319438) that confers a S34Y substitution. While this mutation does not affect the interaction of MYD88 with IRAK1, IRAK4, and Mal, it disrupts MYD88 signaling and NF-κB activation by preventing oligomeric Myddosome complex formation required for downstream signaling (Fig 3B) [31, 32]. The cellular expression level of MYD88 S34Y was comparable to wild type MYD88 (Fig 3C), however the mutated protein failed to activate NF-κB (Fig 3D). More importantly, MYD88 S34Y did not inhibit *Lm* infection (Fig 3E). These findings indicate that MYD88-dependent suppression of *Lm* infection results from strong NF-κB transcriptional activation and currently unknown effector mechanisms.

The ISG screen also revealed *AQP9*, *PKRD2*, *MAP3K14*, and *TRIM14* as potent inhibitors of *Lm* infection, suggesting that these proteins may harbor novel antibacterial activities. Aquaporin 9, encoded by *AQP9*, is a transmembrane channel involved in water and small solute transport [33], whereas PRKD2 and MAP3K14 are kinases implicated in membrane trafficking and immune signaling, respectively [34, 35]. It is currently unclear how expression of these genes blocks *Lm* infection. Among the newly identified anti-listerial ISGs, TRIM14 exhibited the greatest inhibitory activity (Fig 2E). Interestingly, this protein has recently been linked to antiviral defense through several independent mechanisms ([36–38]) but has not been previously implicated in antibacterial immunity.

### Antiviral protein TRIM14 inhibits *Lm* infection in the absence of a transcriptional response

**Fig 4:**
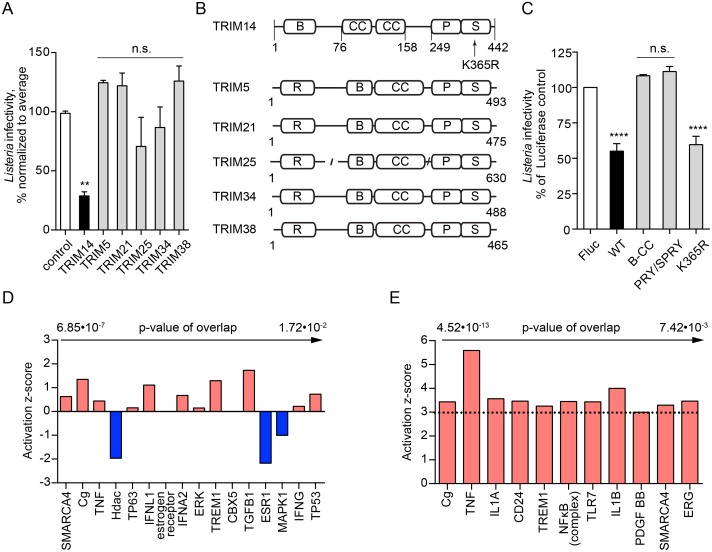
TRIM14 inhibits *Lm* infection in the absence of transcriptional response. (A) Infectivity of wild type *Lm* in *STAT1*-deficient fibroblasts transduced with lentivirus expressing control gene (white bar), *TRIM14* (black bar), *TRIM5*, *TRIM21*, *TRIM25*, *TRIM34*, and *TRIM38* (grey bars) in a large-scale screen. *Lm* infectivity was measured similar to Fig 2C, error bars represent s.d., n = 2 (**, P<0.01; n.s., not significant). (B) Domain architecture of IFN I regulated TRIM proteins. R, RING-type zinc finger domain; B, B box-type zinc finger domain; CC, coiled-coil domain; P S, PRY/SPRY domain. (C) Infectivity of wild type *Lm* in *STAT1*-deficient fibroblasts transduced with lentivirus expressing Fluc (white bar), wild type TRIM14 (WT, black bar), only B-box and coiled-coil domains of TRIM14 (B-CC, grey bar), only PRY/SPRY domain of TRIM14 (PRY/SPRY, grey bar), and TRIM14 K365R mutant (K365R, grey bar). *Lm* infectivity was measured similar to Fig 2C, error bars represent s.d., n = 3 (****, P<0.0001; n.s., not significant). (D) Upstream regulators identified by Ingenuity Pathway Analysis for TRIM14-regulated genes, only top regulators by p-value of overlap are shown. (E) Upstream regulators identified by Ingenuity Pathway Analysis for genes, regulated by *Lm* infection in *STAT1*-deficient fibroblasts, sorted by p-value of overlap with a cutoff of z-score≥3.

TRIM14 is a member of the tripartite motif-containing (TRIM) gene superfamily that includes proteins involved in innate immunity, transcriptional regulation, cell proliferation, and apoptosis [39]. While several family members exhibit anti-viral functions [40], their role in bacterial pathogenesis remains poorly understood. We found that expression of IFN-inducible TRIM5, TRIM21, TRIM25, TRIM34, and TRIM38 had no effect on *Lm* infection (Fig 4A), suggesting that TRIM14 is a unique anti-bacterial effector among the IFN-stimulated TRIMs. As shown in Fig 4B, domain architecture of TRIM14 is distinct from other family members as it is does not encode the RING E3-ligase domain typically found within the N-terminal tripartite motif, and is therefore likely to function through an alternative mechanism [39]. We next asked if TRIM14-mediated antibacterial activity could be attributed to one of its structural domains. Based on the available crystal structures of truncated TRIM proteins [41, 42], we generated TRIM14 constructs consisting of either the B-box with coiled-coil (residues 1–255) or the PRY/SPRY domain (residues 158–442). Notably, separate domains of TRIM14 had no effect on *Lm* infection (Fig 4C), indicating that the full-length TRIM14 is required for the anti-bacterial activity.

TRIM14 was recently reported to play an important role in IFN and NF-κB activation during viral infection [36, 37]. It associates with the mitochondria wherefrom it links MAVS and NEMO to NF-κB and IRF3-activated transcription [37]. It has also been shown to positively regulate type I IFN signaling by inhibiting cGAS degradation [36]. However, our studies were performed in *STAT1*-deficient fibroblasts that cannot be activated by IFN, suggesting that the anti-*Lm* activity of TRIM14 is not associated with this ascribed function. Furthermore, a critical lysine in TRIM14, K365, was shown to be required for IFN activation by TRIM14 in mitochondria [37]. However, we found that K365 was not necessary for the antibacterial function of TRIM14 (Fig 4C). Finally, ectopic expression of TRIM14 in *STAT1*-deficient fibroblasts induced the expression of 36 genes by over 2-fold and only 5 genes had greater than 5-fold increase compared to over 100-fold increase in TRIM14. Ingenuity Pathway Analysis failed to identify possible upstream transcriptional regulators (Fig 4D and S3 Table), and the observed transcriptional response did not exhibit an NF-κB or IRF3 signature as would be predicted if TRIM14 regulated MAVS and NEMO as previously reported.

To then determine if TRIM14 functioned through a transcription-independent mechanism during infection, we compared host mRNA produced during *Lm* infection (6 hours) in cells expressing TRIM14 or luciferase as a control. *Lm* infection altered expression of hundreds of genes in luciferase-expressing cells (S4 Table). As expected, TNFα, NF-κB and IL1A were among the strongest predicted upstream regulators (Fig 4E and S4 Table). Interestingly, expression of TRIM14 did not alter the host transcriptional response to *Lm* infection (S4 Table), further suggesting that TRIM14 has a direct anti-bacterial function in host cells. Taken together, these results indicate that the gain-of-function ISG screening technique can resolve direct mechanisms of inhibition of bacteria, similar to what has been demonstrated for viruses [17].

### High affinity immunoglobulin receptor FcγRIa increases *Lm* infection independently of the canonical host *Lm* internalization receptors

**Fig 5:**
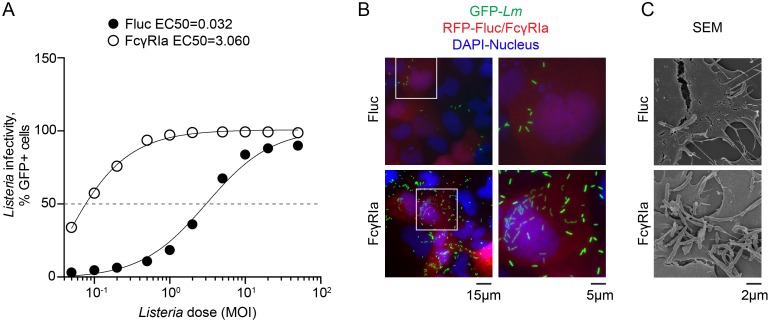
FcγRIa induces a robust *Lm* infection. (A) U-2 OS cells transduced with lentivirus expressing Fluc or FcγRIa were infected with increasing MOI of wild type GFP-expressing *Lm* for 12 h after initial infection. Infectivity was measured by flow cytometry. Dose-response curves were fitted to a sigmoidal model using GraphPad Prism software. (B)(C) Fluorescent microscopy (B) and scanning electron microscopy (C) of U-2 OS cells transduced with lentivirus co-expressing TagRFP and Fluc (*upper*) or FcγRIa (*lower*) and infected with GFP-expressing wild type *Lm* for 5.5 h (B) and 7.5 h (C), following 1.5 h of initial infection.

In addition to anti-bacterial ISGs, we identified a high affinity immunoglobulin receptor FcγRIa (CD64) as an enhancer of *Lm* infection. A dose-response experiment indicated that FcγRIa potentiated *Lm* infectivity by over 100-fold (Fig 5A). Consistent with the flow cytometry measurements, a greater number of individual bacteria were found in the cytoplasm of FcγRIa-expressing cells (Fig 5B) and FcγRIa increased the total number of cell surface protrusions emanating from infected cells (Fig 5C).

**Fig 6:**
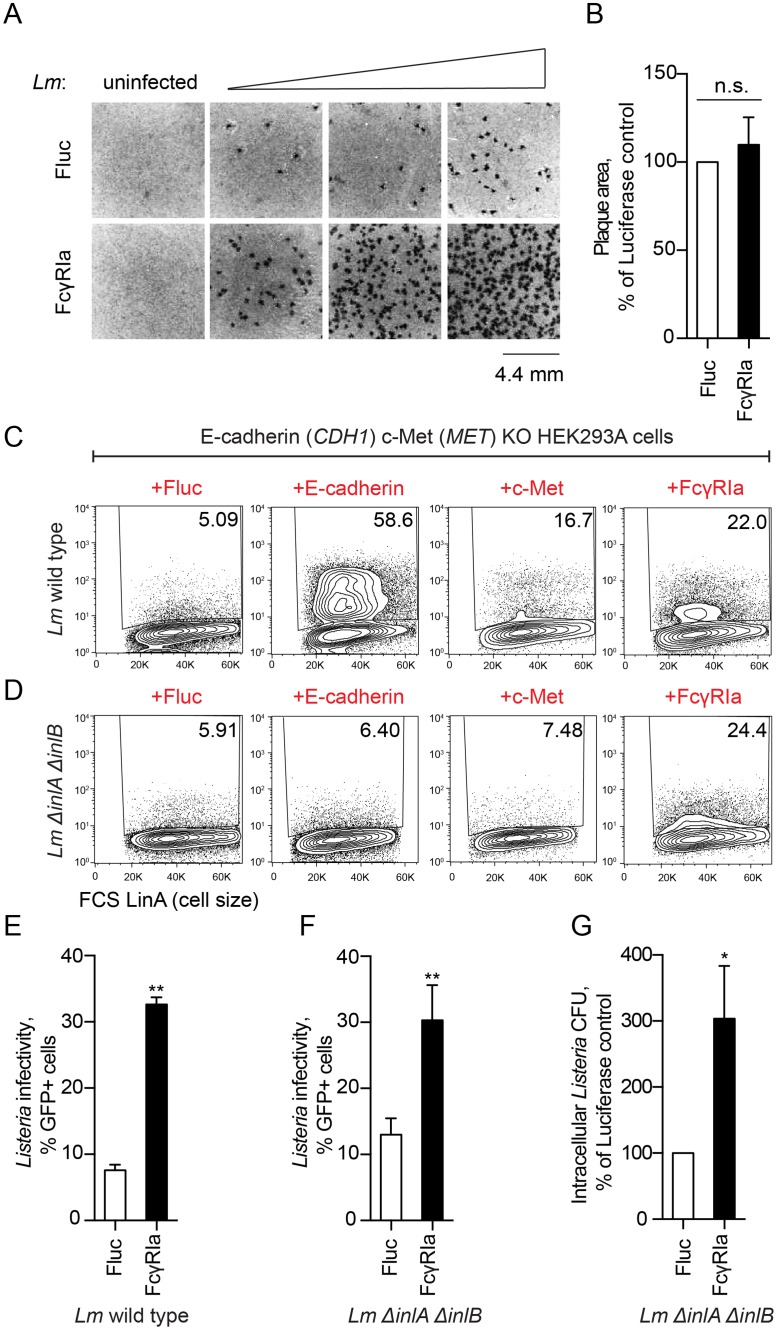
FcγRIa increases *Lm* invasion independently of known *Lm* internalization receptors. (A) Confluent monolayers of HEK293A cells transduced with lentivirus expressing Fluc (*upper*) or FcγRIa (*lower*) were infected with wild type *Lm* and stained for bacteria with (3-(4, 5-dimethylthiazolyl-2)-2,5-diphenyltetrazolium bromide (tetrazolium MTT) 30 h after initial infection. Non-infected controls shown on the left, samples infected with increasing amounts of *Lm* shown left to right. A representative field of each sample is shown. (B) Area of individual plaques obtained in (A) was quantified by using ImageJ software, and presented normalized to Fluc control. Error bars represent s.d., n = 3 independent experiments. Statistical significance was determined by t-test (n.s., not significant). (C-D) Representative flow cytometry plots showing wild type *Lm* (C) or *Lm* Δ*inlA*Δ*inlB* (D) infection of *CDH1/MET*-deficient HEK293A (clone P4E4) transduced with lentivirus co-expressing TagRFP and Fluc, c-Met, E-cadherin or FcγRIa as indicated. Values in the upper right corner of each plot indicate the percentage of GFP-positive cells in the total RFP-positive cell population. (E-G) Infectivity of wild type *Lm* (WT) (E) and *Lm* Δ*inlA*Δ*inlB* (F, G) in *CDH1/MET*-deficient HEK293A cells transduced with lentivirus expressing Fluc (white bars) or FcγRIa (black bars). *Lm* infectivity was measured by flow cytometry (E, F) or as a CFU number of surviving *Lm* in a gentamicin protection assay and normalized to Fluc control (G). Error bars represent s.d., n = 3. Statistical significance was determined by t-test (*, P<0.05; **, P<0.01).

Because FcγRIa is a cell surface expressed protein, we hypothesized that it may enhance *Lm* infection by promoting primary internalization into host cells or secondary cell-to-cell spread. To distinguish between these possibilities, we visualized *Lm* infection foci, which are formed from *Lm* invasion of a single host cell followed by rapid cell-to-cell transmission. Cell monolayers were infected with very low doses of *Lm* (at MOI 0.015, 0.05, 0.1) and the formation of foci was evaluated 30 hours post infection (see Materials and Methods). Cellular expression of FcγRIa increased the total number of infection foci compared to control (Fig 6A). However, the diameter and surface area of individual foci were not altered in presence of FcγRIa (Fig 6B). Thus, FcγRIa enhances efficiency of primary *Lm* invasion, yet has little effect on secondary cell-to-cell spread.

We next asked if FcγRIa potentiated *Lm* entry by coordinating interactions with the host *Lm* internalization receptors E-cadherin or c-Met [43]. We introduced frameshift mutations into *CDH1* (encoding E-cadherin) and *MET* (encoding c-Met) by CRISPR/Cas9 resulting in non-coding genetic disruption of these loci (S2A–S2F Fig). As expected, the invasive capacity of *Lm* was significantly attenuated in *CDH1/MET*-deficient cells (S3G Fig), which could be restored by ectopic expression of either *CDH1* or *MET* (Fig 6C). Remarkably, ectopic expression of FcγRIa in *CDH1/MET*-deficient cells increased *Lm* infection to levels comparable with *MET* complementation (Fig 6C and 6E). In addition, mutant *Lm ΔinlAΔinlB* lacking the invasins InlA and InlB that directly bind host surface proteins E-cadherin and c-Met, respectively, readily infected *CDH1/MET*-deficient cells expressing FcγRIa (Fig 6D, 6F and 6G). Therefore, FcγRIa supports bacterial uptake independently of “classic” host *Lm* internalization receptors E-cadherin and c-Met as well as bacterial invasins InlA and InlB.

### Reconstitution of Fcγ receptor function in non-phagocytic cells

Fcγ receptors bind the Fc (antigen non-specific) region of IgG antibodies produced as a part of adaptive response to infection in mammals. The human Fcγ receptor family includes activating receptors FcγRIa, FcγRIIa, FcγRIIc, FcγRIIIa and FcγRIIIb, as well as an inhibitory receptor FcγRIIb. Crosslinking of activating Fcγ receptors by IgG typically results in the phagocytosis of opsonized particles and cellular activation, facilitating destruction of the pathogens and induction of inflammation, respectively [44]. In humans, FcγRIa is constitutively expressed on monocytes and macrophages, and its expression is upregulated by type I and II interferons and other signaling molecules, such as IL-10 [45]. It consists of three extracellular immunoglobulin (Ig)-like domains, a single transmembrane domain, and a short cytoplasmic tail that does not contain any known signaling motifs. During receptor engagement with IgGs, FcγRIa recruits the accessory immunoreceptor tyrosine-based activation motif (ITAM)-containing γ-chain (FcεRIg). Clustering of the FcγRIa with γ-chain triggers intracellular signaling cascades involving Syk and Src family kinases necessary for FcγRIa-mediated particle phagocytosis [46, 47]. Additionally, FcγRIa has been shown to interact with FcγRIIa, using its ITAM-motif to signal in the absence of the γ-chain [48].

**Fig 7:**
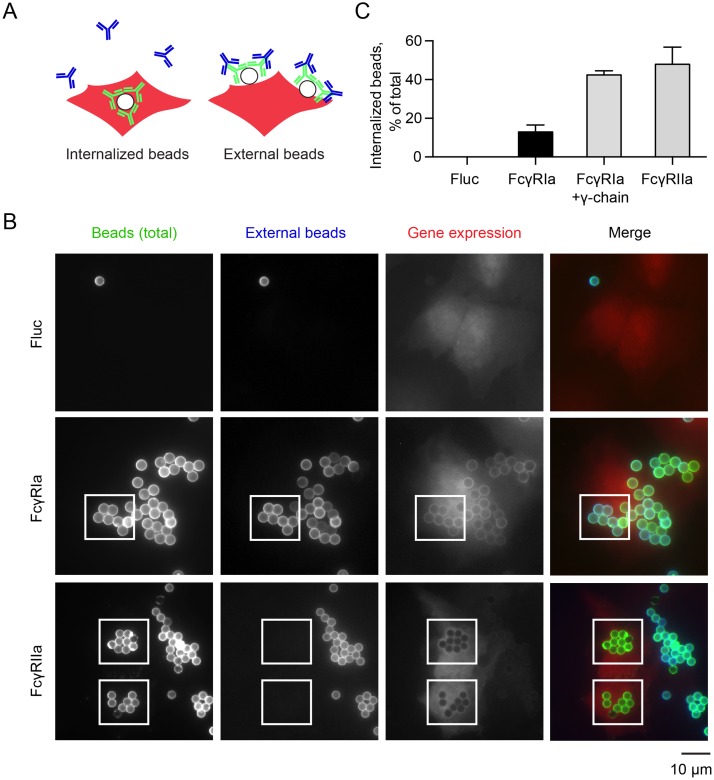
Developing a cellular model of FcγRIa function. (A) Diagram illustrating the phagocytic assay used to reconstitute FcγR function using Alexa Fluor 488 IgG (green) and DyLight 405 (blue) IgG-labeled polystyrene beads. (B) Representative fluorescence microscopy images of U-2 OS cells transduced with lentivirus co-expressing TagRFP and Fluc, FcγRIa, or FcγRIIa, incubated with Alexa Fluor 488 IgG-opsonized beads (green) for 1.5 h, followed by secondary DyLight 405 IgG labeling (blue) of external beads. (C) Quantification of phagocytosed IgG-coated beads. Error bars represent s.d., 160 cells were counted for each of three independent experiments.

To compare the mechanism of *Lm* invasion to the classic IgG-coated particle uptake though FcγRIa alone in the absence of possible crosstalk with other Fcγ receptors [44, 48], we developed a model of Fc-receptor functions in a non-phagocytic cell type [46, 49–51]. We first reconstituted IgG-coated particle internalization via FcγRIa. U-2 OS cells were transduced with a lentivirus expressing FcγRIa, or Fluc as a negative control. Latex beads were coated with human IgG and labeled with anti-human secondary antibody conjugated to Alexa Fluor 488 (green). The IgG opsonized particles were incubated with U-2 OS cells for 1.5 h at 37°C and then shifted to 4°C to inhibit further uptake. Cell-surface bound beads were differentiated from internalized beads by incubating samples with anti-human secondary antibody conjugated to DyLight 405 (blue) without cell permeabilization (Fig 7A). Under these conditions, internalized beads are protected from the secondary antibody and are visualized as green beads by fluorescence microscopy. In contrast, surface-bound beads are labeled with both green and blue secondary antibodies.

As expected, luciferase-expressing U-2 OS cells showed no interaction with IgG-coated beads. In contrast, FcγRIa recruited IgG-beads to the cell surface, but revealed low levels of bead internalization (Fig 7B and 7C). This may be anticipated since U-2 OS cells do not express endogenous γ-chain (FcεR1g). Indeed, co-expression of FcγRIa with the γ-chain (FcεR1g) fully reconstituted FcγRIa-mediated internalization of IgG-coated beads (Fig 7C). We also cloned and tested another Fcγ receptor–FcγRIIa–a low-affinity immunoglobulin receptor that possesses its own internal ITAM motif and therefore, does not require interaction with the γ-chain for particle internalization. FcγRIIa mediated similar high levels of IgG-coated bead phagocytosis (Fig 7B and 7C). Thus, we have established a robust and simplified cellular system to study the function of individual human Fcγ receptors in context of both particle opsonization and pathogenic *Lm* infection.

### FcγRIa mediates *Lm* internalization independent of γ-chain and pathogen opsonization

**Fig 8:**
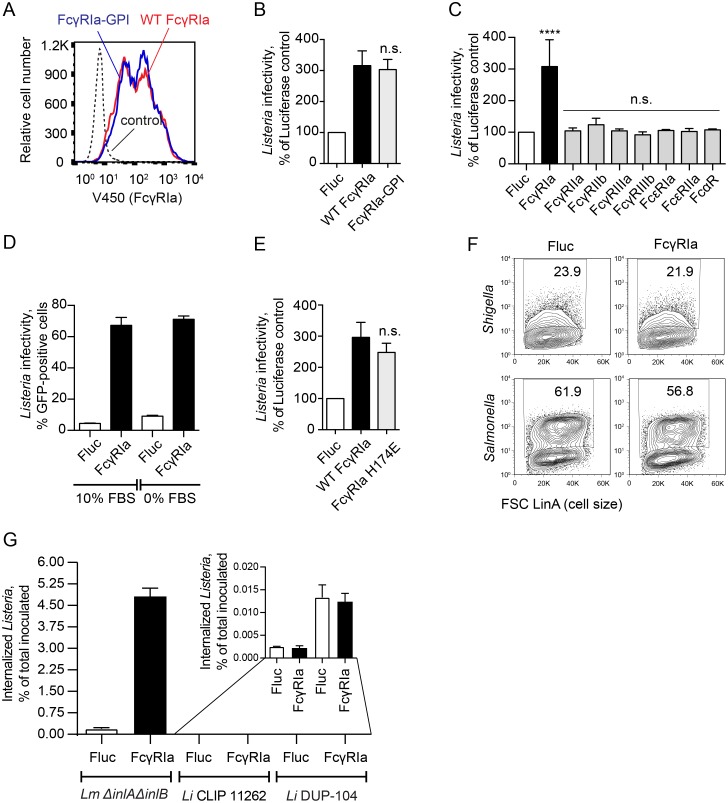
FcγRIa increases *Lm* infection independently of the γ-chain and opsonization by IgG. (A) Flow cytometric analysis of surface expression of wild type FcγRIa and FcγRIa-GPI in lentiviral transduced HEK293A cells. (B) Infectivity of *Lm* Δ*inlA*Δ*inlB* in HEK293A cells transduced with lentivirus expressing Fluc (white bar), wild type FcγRIa (black bar), or FcγRIa-GPI (grey bar). *Lm* infectivity was measured as in Fig 2C, error bars represent s.d., n = 3 (n.s., not significant, as compared to wild type FcγRIa). (C) Infectivity of wild type *Lm* in *CDH1/MET*-deficient HEK293A cells (clone P4E4) transduced with lentivirus expressing the indicated Fc-receptors. *Lm* infectivity was measured as in Fig 2C, error bars represent s.d., n = 3 (****, P<0.0001, n.s., not significant). (D) Infectivity of wild type *Lm* in U-2 OS cells stably expressing Fluc (white bars) or FcγRIa (black bars) in DMEM media, containing 10% FBS (*left*) or FBS-free media (*right*). *Lm* infectivity was measured after 2 h initial invasion time and 3 h infection, by flow cytometry and represented as a percentage of GFP-positive cells, n = 3, s.d. (E) Infectivity of wild type *Lm* in HEK293A cells transduced with lentivirus expressing Fluc (white bar), wild type FcγRIa (black bar), or H174E mutant FcγRIa (grey bar). *Lm* infectivity was measured as in Fig 2C, error bars represent s.d., n = 3 (n.s., not significant, as compared to wild type FcγRIa). (F) Representative flow cytometry plots of *Shigella flexneri* (*top*) and *Salmonella* Typhimurium (*bottom*) infections in *STAT1*-deficient fibroblasts transduced with lentivirus co-expressing TagRFP and Fluc (*left*) or FcγRIa (*right*). Also see S3 Fig. (G) Invasion of *Lm ΔinlAΔinlB*, *L*. *innocua* CLIP 11262 and *L*. *innocua* DUP-104 in HEK293A cells transduced with lentivirus expressing Fluc (white bars) or FcγRIa (black bars). Invasion efficiency was measured as a number of *Listeria* colony forming units (CFU) surviving in a gentamicin protection assay, divided by the total CFU used for infection. Error bars represent s.d., n = 3. MOI = 10. Statistical significance was determined by t-test (n.s., not significant). (Inset) Expanded view.

As shown in Fig 5A, *Lm* readily invaded U-2 OS cells expressing FcγRIa. Surprisingly, this phenotype did not require co-expression of the ITAM-containing γ-chain, suggesting that *Lm* internalization by FcγRIa occurs through a distinct mechanism compared to IgG-coated particle uptake. It has been previously reported that FcγRIa interacts with the γ-chain exclusively through the transmembrane domain [52]. We therefore asked if this region of FcγRIa was necessary for *Lm* internalization. We targeted the extracellular Ig-like domains of FcγRIa to the cell surface via a GPI-anchor signal of LFA-3 (FcγRIa-GPI) [53]. This chimeric protein was expressed on the cell surface similar to the wild type FcγRIa (Fig 8A). Notably, as shown in Fig 8B, FcγRIa-GPI induced the same level of *Lm* infection as the wild type protein (3.03 fold), further confirming that FcγRIa does not interact with the γ-chain during *Lm* internalization. Additionally, FcγRIa does not require interaction with any other signaling protein through the transmembrane domain.

The ability of *Lm* to be internalized by FcγRIa in the absence of the signaling γ-chain suggested that the recognition of *Lm* might also occur independently of IgG opsonization. Several lines of evidence support this conclusion. First, *Lm* infection was not enhanced by expression of other members of the Fc receptor family (Fig 8C), including FcγRIIa that, as shown in Fig 7C, was able to internalize IgG-coated beads. Second, FcγRIa potentiated *Lm* invasion in serum-free (and therefore, IgG-free) conditions (Fig 8D). Third, reducing the FcγRIa affinity for all types of IgG up to 100-fold by introducing an H174E mutation in the D2 Ig-like domain [54] did not affect its ability to enhance *Lm* infection (Fig 8E). Finally, FcγRIa had no effect on the infection rate of other intracellular bacteria *Shigella flexneri* or *Salmonella* Typhimurium (Fig 8F and S3A and S3B Fig). Therefore, internalization through FcγRIa is independent of non-specific pathogen opsonization with serum IgG. Together, these data indicate that *Lm* invades cells independently of the well-established route of phagocytosis, which involves IgG opsonization and ITAM-mediated intracellular signaling through the FcγRIa-γ-chain complex.

### Nonpathogenic *Listeria innocua* is not internalized by FcγRIa

Since our data revealed a novel mechanism of FcγRIa function that included *Lm* internalization independent of IgG opsonization, we hypothesized that *Lm* might express a cell surface factor required for *Lm*-FcγRIa interaction. To determine if this molecule was a general feature of *Listeria* genus or specific to pathogenic *Lm*, we assessed the ability of FcγRIa to confer invasiveness to a closely related but non-pathogenic species *L*. *innocua*. While *L*. *innocua* strains are genetically heterogeneous and may encode various combinations of genes shared with *Lm* [55, 56], *L*. *innocua* CLIP 11262 has been fully sequenced and annotated. It has been shown to lack all major genes required for *Lm* pathogenesis (*inlA*, *inlB*, *hly*, *actA*, etc.) [57]. As show in Fig 8G, in the absence of FcγRIa, invasion rates of non-pathogenic *L*. *innocua* CLIP 11262 were 78.43-fold lower than those of invasion-deficient *Lm ΔinlAΔinlB*. Importantly, while FcγRIa expression increased internalization of *Lm ΔinlAΔinlB*, it did not allow invasion of the CLIP 11262 strain (Fig 8G). Similarly, FcγRIa did not increase internalization of an unsequenced *L*. *innocua* strain DUP-104 (Fig 8G), suggesting that the bacterial ligand is specific to *Lm* and is not shared with non-pathogenic *Listeria* strains.

### FcγRIa and *Lm* infection of macrophages *in vitro*

**Fig 9:**
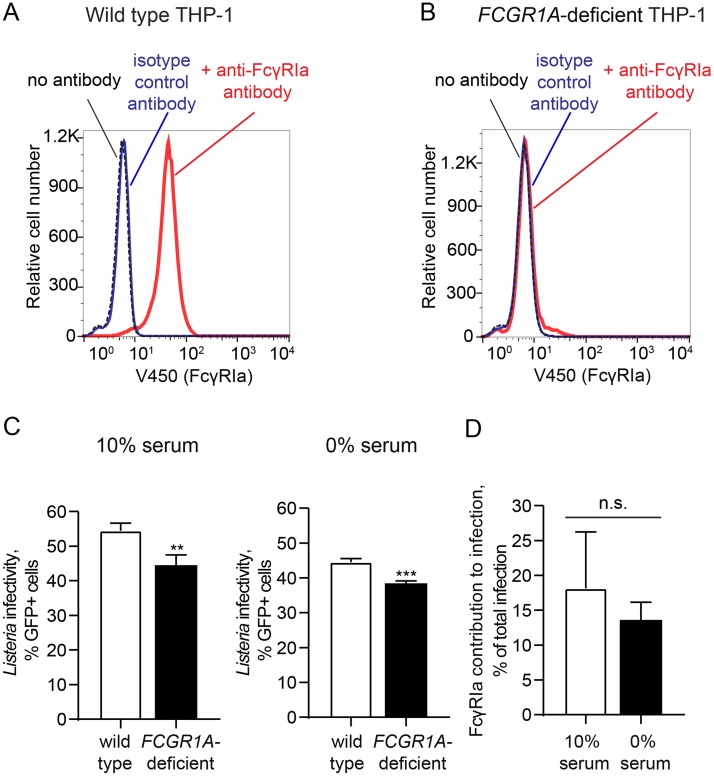
FcγRIa and *Lm* infection of phagocytic cells. (A-B) Surface expression of FcγRIa in wild type (A) and *FCGR1A*-deficient (B) THP-1 cells was analyzed by flow cytometry. (C) Infectivity of wild type *Lm* in wild type (white bars) and *FCGR1A*-deficient (black bars) THP-1 cells in 10% FBS/RPMI medium (*left*) or serum-free RPMI (*right*). Cells were infected (MOI = 5) for an initial 90 min period, when gentamicin was added and infection was allowed to proceed for an additional 6 h prior to collection. *Lm* infectivity was measured as percentage of GFP-positive cells, n = 3, error bars represent s.d., significance was determined by t-test for each condition (**, P<0.01; ***, P<0.001). (D) Relative contribution of FcγRIa to *Lm* infection in THP-1 cells in 10% FBS/RPMI and serum-free RPMI medium, calculated as described in Materials and Methods, n = 3, error bars represent s.d. (n.s., not significant).

To determine the contribution of FcγRIa to *Lm* infection in a naturally phagocytic human cell type that expresses endogenous FcγRIa, we disrupted cell surface expression of *FCGR1A* in THP-1 human monocytes using a lentiviral CRISPR/Cas9 system [58] (Fig 9A and 9B). *Lm* infected 54.45 ± 2.19% wild-type THP-1 cells compared to 44.48 ± 2.98% of *FCGR1A*-deficient cells (Fig 9C) representing a statistically significant decrease in infection (p = 0.0095, n = 3). These data suggest that *Lm* is internalized through multiple pathways with 18.13 ± 8.1% (n = 3) of the total host cell infection mediated by FcγRIa (Fig 9D). To then determine if the observed decrease in *Lm* infection was due to the newly defined mechanism of FcγRIa-*Lm* interaction described above rather than a general defect in IgG-coated pathogen internalization, we performed experiments in serum-free conditions. A significant reduction in *Lm* infection was observed in *FCGR1A*-deficient THP-1 (44.52 ± 1% infected wild type cells compared to 38.44 ± 0.69% of *FCGR1A*-deficient cells; p = 0.0010, n = 3) (Fig 9C) with a 13.63 ± 2.52% (n = 3) relative contribution of FcγRIa under these conditions (Fig 9D). Thus, endogenous FcγRIa contributes to *Lm* invasion of phagocytic monocytes independently of IgG opsonization similar to what was observed in the reconstituted cellular system.

### Host specificity of FcγRIa-mediated *Lm* internalization

**Fig 10:**
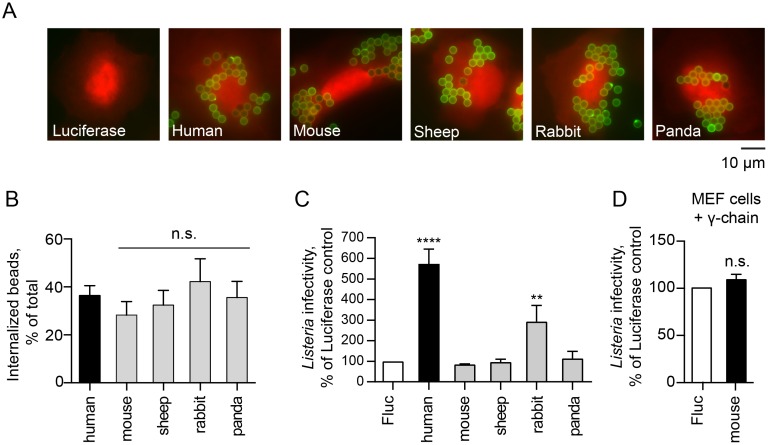
FcγRIa-mediated *Lm* invasion exhibits host species tropism. (A) Representative fluorescence microscopy images of U-2 OS cells transduced with lentivirus co-expressing TagRFP and Fluc or FcγRIa from indicated species incubated with Alexa Fluor 488 IgG-opsonized beads (green) for 1.5 h. (B) Quantification of phagocytosed human IgG-coated beads in U-2 OS cells transduced with lentivirus expressing γ-chain and FcγRIa of indicated species. Error bars represent s.d., 40 cells were counted for each of the four independent experiments (n.s., not significant). (C) Infectivity of *Lm ΔinlAΔinlB* in HEK293A cells transduced with lentivirus expressing Fluc (white bar), human FcγRIa (black bar) or FcγRIa from indicated species (grey bars). *Lm* infectivity was measured as in Fig 2C, n = 3, error bars represent s.d. (****, P<0.0001). (D) Infectivity of wild type *Lm* in MEFs transduced with lentivirus expressing human γ-chain and Fluc or murine FcγRIa. *Lm* infectivity was measured as in Fig 2C, error bars represent s.d., n = 3, statistical significance was determined by t-test prior to normalization (n.s., not significant).

Having established that human FcγRIa enhances *Lm* infection, we next asked whether mammalian FcγRIa orthologs exhibit similar functions. Species-specific FcγRIa coding sequences were commercially synthesized, and included (1) mouse (naturally resistant to oral *Lm* infection), (2) sheep and rabbit (known to be susceptible to *Lm*), and (3) panda (uncharacterized susceptibility to *Lm* infection). All FcγRIa orthologs were expressed on the cell surface of U-2 OS cells as determine by IgG-coated latex bead binding assays (Fig 10A). We then co-expressed these receptors with the γ-chain and tested whether they were fully functional in human cells by measuring the rates of IgG-opsonized particle internalization. All tested FcγRIa induced similar levels of IgG-bead phagocytosis (Fig 10B), suggesting that they were indeed functioning as internalization receptors for opsonized particles. Next, we assessed the ability of non-primate FcγRIa to potentiate internalization of *Lm*. FcγRIa of mouse, sheep and panda failed to enhance *Lm* infection (Fig 10C). Moreover, murine FcγRIa did not affect *Lm* infection even when co-expressed with the γ-chain in murine cells (Fig 10D). Unexpectedly, rabbit FcγRIa was found to potentiate *Lm* internalization in the absence of the γ-chain (Fig 10D). Rabbit is a natural host for *Lm* and exhibits severe listeriosis upon infection [59]. Analysis of the multiple sequence alignment of FcγRIa from these species did not pinpoint a single residue or a motif that was common between human and rabbit yet divergent from other FcγRIa proteins tested, suggesting a more complex interaction between host and pathogen molecules (S4 Fig). Nevertheless, these data indicate that FcγRIa-*Lm* interaction is not only pathogen-specific (Fig 8F), but also demonstrates host protein tropism.

## Discussion

The host type I interferon response is stimulated by numerous bacterial pathogens. However, the roles of individual ISGs in restricting bacterial infection are not well characterized. To address this gap in the knowledge of IFN biology, we adapted a gain-of-function screening approach to identify cellular regulators of *Lm* infection among approximately 350 type I ISGs. The screen revealed strong cell-autonomous inhibitors of *Lm* infection, such as TRIM14, AQP9, MYD88, UNC93B1 and MAP3K14. Interestingly, we also identified the human immunoglobulin receptor FcγRIa as an enhancer of *Lm* internalization, suggesting an intriguing possibility that bacterial pathogens have evolved virulence factors to directly hijack the IFN response system.

We identified type I IFN-stimulated inhibitors of *Lm* infection that function through the upregulation of complex gene expression profiles (e.g. MYD88) and/or through direct anti-microbial mechanisms (e.g. TRIM14). These ISGs may contribute to the regulation of *Lm* in a wide variety of tissue environments. For example, upregulation and activation of MYD88 in TLR-expressing lymphocytes would result in the expression of NF-κB-regulated genes with broad antibacterial activity. Our data indeed suggest that a MYD88-induced transcription program suppresses *Lm* infection through NF-κB activation (Fig 3C–3E). Notably, *Lm* has been previously reported to counteract host defense systems, including interfering with NF-κB activation, thus dampening the overall inflammatory response to infection [60]. Our findings now indicate that inhibition of NF-κB by *Lm* may protect the pathogen from previously unknown cell-autonomous immune mechanisms. Further studies are needed to confirm this speculation. Another strong inhibitory ISG, TRIM14 is widely expressed throughout the body, including organs targeted by *Lm*, such as intestine and liver [37, 61]. However, this is not the first study to implicate TRIM14 in anti-microbial defense. Recent studies characterized TRIM14 as an antiviral protein that activates both NF-κB and type I IFN through bridging MAVS and NEMO proteins as well as inhibiting cGAS degradation [36, 37]. Interestingly our data support an alternative mechanism for the function of TRIM14. We found that TRIM14 inhibited *Lm* infection in cells with defective IFN responses and that ectopic expression of TRIM14 did not alter the host transcriptional profile induced by *Lm* (Fig 4). Further studies are needed to reveal the precise inhibitory mechanisms of TRIM14 as well as other antilisterial ISGs including PRKD2, AQP9, and MAP3K14 identified here.

Perhaps the most surprising discovery of this work is that the immunoglobulin receptor FcγRIa mediates *Lm* uptake, contributing to *Lm* invasion of phagocytic monocytes and macrophages. This finding is particularly insightful since these cells are not only an important target of *Lm* infection, but also aid the transmission of *Lm* to peripheral tissues during infection [62]. Currently, the precise molecular mechanisms of *Lm* internalization in phagocytic cells have not been characterized in detail and are believed to be mediated by C3bi and C1q complement receptors and phagocyte scavenger receptors [63, 64]. However, our studies now suggest that *Lm* hijacks an alternative pathway to invade phagocytic cells through an immunoglobulin-independent interaction with FcγRIa. While studies presented here have elucidated many key aspects of the internalization process (see below), several questions remain unanswered: (1) what is the nature of the IgG-independent interaction between *Lm* and FcγRIa resulting in *Lm* uptake by the host cells, (2) what is the cellular mechanism of FcγRIa-mediated *Lm* internalization; and, finally, (3) what are the consequences of this interaction for both pathogen and host in terms of pathogen proliferation and disease outcomes.

In this study, we provide compelling evidence that *Lm* is internalized by FcγRIa independently of IgG opsonization (Fig 8). The most direct explanation for these findings is that *Lm* directly engages FcγRIa at the surface of immune cells. However, the identity of the bacterial surface protein involved in the interaction remains unclear. FcγRIa was unable to induce invasion of a non-pathogenic *L*. *innocua* (Fig 8G), thus narrowing down the search for the ligand to a small subset of *Lm*-specific surface proteins [57]. Importantly, we have ruled out the involvement of the most well-characterized *Lm* invasion factors—internalins InlA and InlB (Fig 6), known to act individually or in concert to trigger *Lm* entry into a wide variety of non-phagocytic cells [43, 65]. Other *Lm*-specific proteins previously implicated in the entry of *Lm* into target cells, virulence factor ActA, as well as less-characterized Vip, LapB, and Auto may be involved in FcγRIa interaction [43, 66–68]. Both biochemical studies on candidate bacterial surface proteins as well as unbiased genetic screens will help determine if the FcγRIa ligand is a known invasion protein or a novel factor previously not implicated in *Lm* infection. Notably, a similar IgG-independent interaction has been demonstrated between FcγRIa and *Escherichia coli* K1 [69]. These bacteria have been shown to invade macrophages as a result of the interaction of the bacterial Outer membrane protein A (OmpA) with FcγRIa. It therefore appears that targeting IgG-independent functions of FcγRIa may be a general pathogenic strategy to evade immune clearance during systemic infection.

While work presented in this study clearly indicates that FcγRIa facilitates entry of *Lm* into host cells, the cellular signaling mechanisms required for this process remain unknown. Since FcγRIa itself does not contain any known signaling motifs, the FcγRIa-mediated phagocytosis of IgG-coated particles requires receptor interaction with the ITAM-domain containing γ-chain, which in turn mediates downstream signaling, triggering cytoskeleton rearrangement and particle internalization [46, 70]. Interaction of FcγRIa with the γ-chain occurs exclusively through the transmembrane domain of the receptor [52]. However, we found that GPI-anchored FcγRIa preserved its ability to internalize *Lm* in the absence of the transmembrane domain (Fig 8B), indicating that both transmembrane and intracellular domains of FcγRIa were dispensable for this process. Thus, our data reveal the existence of an alternative non-canonical mechanism of FcγRIa internalization. It is currently unclear if FcγRIa-mediated uptake of *Lm* resembles the extensively characterized mechanism of *Lm* uptake by non-phagocytic cells through E-cadherin and c-Met receptors. *Lm*-induced clustering of these receptors leads to the recruitment of clathrin-mediated endocytosis machinery, actin cytoskeleton organization, and modulation of the phosphoinositide metabolism at the site of bacterial adhesion, resulting in the engulfment of the pathogen by zipper-like mechanism [71]. It will be of interest to define the involvement of actin, clathrin, and intracellular signaling pathways in the FcγRIa-mediated *Lm* entry.

It is intriguing to speculate on the potential role of FcγRIa in *Lm* pathogenesis. We found that a small but reproducible percentage of THP-1 infection (~18%) was dependent on cell surface expression of endogenous FcγRIa (Fig 9). Therefore, our data reveal the existence of at least two distinct pathways for *Lm* invasion including a canonical phagocytic pathway and a novel FcγRIa-mediated pathway described here. We hypothesize that *Lm* may have evolved surface molecules to engage the FcγRIa internalization pathway and bypass cell-mediated killing induced by other phagocytic routes of internalization. Consistent with this idea, *Lm* did not specifically engage the major phagocytic Fcγ receptor FcγRIIa involved in pathogen clearance in neutrophils and monocytes (Fig 8C). In addition, previous studies have demonstrated fundamental differences in intracellular signaling pathways, receptor trafficking, antigen presentation, and kinetics of oxidative burst triggered by high-affinity IgG receptors (FcγRIa) compared to low affinity receptors (FcγRIIa) [72]. Thus, the ability of *Lm* to exploit the high affinity IgG receptor rather than being phagocytosed through the canonical opsonization pathway by FcγRIIa, may provide an opportunity for invaded *Lm* to produce phagosome rupture factors and escape into the cytoplasm. While this scenario has not yet been substantiated *in vivo*, the challenge for future studies will be to examine *Lm* internalization by FcγRIa in primary human cells revealing the role of FcγRIa in *Lm* pathogenesis.

In conclusion, our flow cytometry based screening approach not only uncovered type I IFN stimulated suppressors of *Lm* infection but also revealed a novel *Lm* uptake pathway, which may play an important role in human *Lm* infection and disease pathogenesis. This work also opens up new experimental avenues to examine the role of IFNs, and potentially other immune modulatory transcriptional programs, in the pathogenesis of a wide range of bacterial species, including both intracellular bacteria that replicate in either vacuoles or cytoplasmic environment, and extracellular bacteria that may be affected by secreted ISGs.

## Materials and Methods

### Bacterial strains

*Listeria monocytogenes* 10403s constitutively expressing Green Fluorescent Protein (GFP), *L*. *monocytogenes* DP-L2319 (10403s *Δhly ΔplcA ΔplcB*), and DP-L3078 (10403s *ΔactA)* strains were a gift from Dan Portnoy (UC Berkeley). *L*. *monocytogenes* 10403s Δ*inlA* Δ*inlB*, expressing GFP (LM 131) was kindly provided by Manuel Amieva (Stanford). To generate *Lm* 10403s *Δhly ΔplcA ΔplcB* and *ΔactA* pactA::GFP strains pPL2-GFP construct was chemically transformed into *E*.*coli* SM10, followed by conjugation with the DP-2319 strain. GFPmut2 was PCR amplified from the genomic DNA of *Listeria* strain *LM*124 and then cloned downstream of the actA proximal promoter (200bp upstream) in the pPL2 vector. pPL2 was used to integrate genes at the *tRNA*^Arg^ locus of the *Listeria* chromosome [73].

*L*. *innocua* strains DUP-104 [LCDC 81–861] and BAA-680 (CLIP 11262) (genome sequencing strain) were obtained from ATCC. Additionally, *Shigella flexneri* strain M90T (serotype 5) with pBBRMCS1-GFP plasmid and GFP-expressing *Salmonella* Typhimurium str. SL1344 expressing pBBR1MCS 6Y GFP were used.

### Mammalian cell culture

*STAT1*-deficient fibroblasts (an SV40 large T antigen immortalized skin fibroblast line, kindly provided by Jean-Laurent Casanova, Rockefeller University) were grown in RPMI Medium 1640 (Gibco, Thermo Fisher Scientific), supplemented with 10% Fetal Bovine Serum (FBS) (Gibco, Thermo Fisher Scientific) and non-essential amino acids (NEAA) (Gibco, Thermo Fisher Scientific). HEK293A (Jack Dixon, UC San Diego), HEK293T (Paul Bieniasz, Aaron Diamond AIDS Research Center), U-2 OS (ATCC), and MEF (Charles Rice, Rockefeller University) cells were maintained in Dulbecco's Modified Eagle Medium (DMEM) (Gibco, Thermo Fisher Scientific), supplemented with 10% FBS and NEAA. THP-1 cells (ATCC) were cultured in RPMI Medium 1640, ATCC modification (Gibco, Thermo Fisher Scientific), supplemented with 10% FBS and NEAA.

### DNA constructs

cDNA for human *FCGR1B*, *FCGR2B*, *FCGR3A*, *FCGR3B*, *FCER1A*, *FCER2A*, *FCER1G*, *FCAR1* were obtained from the Ultimate ORF Clones (96-well plate) collection (Life Technologies) as Gateway-compatible pENTR clones. cDNA for human *FCGR2A* was a gift from Dr. Eric Hansen (UTSW). These genes were amplified by PCR with primers encoding attB sites. Polymerase chain reaction (PCR) products were purified with the QIAquick PCR Purification Kit (Qiagen) and then recombined into a pDONR221 vector using BP Clonase II Enzyme mix (Life Technologies). BP reactions were transformed into chemically competent DH5a *Escherichia coli*, and colonies verified by sequencing. Resulting pENTR clones were further recombined into a pTRIP.CMV.IVSb.ires.TagRFP Destination vector [17] using LR Clonase II Enzyme mix (Life Technologies). LR reactions were transformed into DH5α cells and verified by sequencing.

pLenti CMV Puro DEST (w118-1) for generation of stable cell lines was a gift from Eric Campeau (Addgene plasmid #17452) [74]. *FLUC*, *FCGR1A*, *and FCER1G* (referred to as γ-chain) were introduced using LR Clonase II Enzyme mix (Life Technologies) as described above.

Point mutations and truncations were generated by PCR of the corresponding pENTR clones using a QuikChange II XL Site-Directed Mutagenesis Kit (Agilent) and primers designed according to manufacturer’s instructions. Glycosylphosphatidylinositol (GPI) anchored FcγRIa (previously described in [53] was generated by overlap extension PCR, using *FCGR1A* and *LFA3*, obtained from the Ultimate ORF Clones (96 well plate) collection (Life Technologies), as templates.

Sheep (NM_001139452.1), rabbit (XM_008264510.1), and panda (XM_011217915.1) *FCGR1A* cDNA were codon optimized for expression in human cells using Codon Optimization Tool (Integrated DNA Technologies) and synthesized as gBlocks Gene Fragments (Integrated DNA Technologies) with addition of attB sites. Mouse (NM_010186) *FCGR1A* cDNA was synthesized as a pENTR clone (GeneCopoeia, Inc). Genes were recombined into pDONR221 and subsequently into expression vector pTRIP.CMV.IVSb.ires.TagRFP Destination vectors as described above.

pX335-U6-Chimeric_BB-CBh-hSpCas9n(D10A) was a gift from Feng Zhang (Addgene plasmid # 42335) [75]. LentiCRISPR v2 was a gift from Feng Zhang (Addgene plasmid # 52961) [58].

### Generation of ISG-expressing lentiviral pseudoparticles

Lentiviral pseudoparticles were generated as previously described [17].

### Lentiviral transduction

Lentiviral transduction was performed as previously described [17]. Briefly, cells were seeded in 24-well tissue culture plates at a density of 7x10^4^ cells per well and transduced the following day with lentiviral pseudoparticles via spinoculation at 1,000 x *g* for 45 min in medium containing 3% FBS, 20mM HEPES and 4 μg/ml polybrene. 6 h after spinoculation, pseudoparticle-containing media was removed and replaced with full cell culture medium, containing 10% FBS and NEAA. For subsequent bacterial infection, cells were split 1:2 48h after transduction. For generation of stable expressing cell lines using pLenti CMV Puro DEST (w118-1), cells were transduced with the lentivirus and selected for puromycin resistance for 7 days 48h after transduction.

### Bacterial infection

*Listeria monocytogenes* was inoculated from a frozen stock and grown for 13 h at 30°C in brain–heart infusion media (BHI) (Difco, BD Biosciences) without shaking. 1 ml of bacteria was then washed in phosphate buffer saline (PBS) and resuspended in 1ml of PBS. A 1:10 dilution of the bacterial suspension was used to read the optical density at 600 nm (OD_600_). Bacteria were then added to each well of cells to achieve multiplicity of infection (MOI) of 10, unless otherwise stated, and incubated for 90 min at 37°C, 5% CO_2_ (unless otherwise noted). Culture media was then removed and replaced with media supplemented with 25 μg/ml gentamicin (Quality Biological) and cells incubated at 37°C, 5% CO_2_ for the indicated period of time. *STAT1-*deficient fibroblasts were infected with *Lm* for 6 h, HEK293A –for 4 h, MEF– 3.5 h, THP-1–6 h, unless otherwise stated in figure legend, U-2 OS–see specific figure legends.

*L*. *innocua* infection was performed following a similar protocol with MOI of 10, invasion was measured as described in “Measuring intracellular bacterial burden” (see below) 1 h following 1.5 h initial infection.

For *Lm* infection of THP-1 cells, 8x10^4^ cells were seeded per well in 96-well tissue culture plates in 10% FBS/RPMI or serum-free RPMI. 24 h later later *Lm* infection was performed as described above (MOI = 5). Following 1.5 h initial invasion time, gentamicin-containing media was added to the wells (final concentration 30 μg/ml) and infection was allowed to proceed for 6 h. Contribution of FcγRIa to *Lm* infection in each independent experiment was calculated using the following equation: [(percent infected wild type cells)–(percent infected *FCGR1A*-deficient cells) / [(percent infected wild type cells)] x 100%.

To visualize bacterial infection by epifluorescence microscopy, cells were washed once in PBS, fixed in 3.7% formaldehyde in PBS for 10 min at room temperature. Cells were then washed three times in PBS and incubated for 2 min in 4',6-diamidino-2-phenylindole (DAPI) solution.

*Shigella flexneri* strain M90T was inoculated from a frozen stock and grown overnight at 30°C in BHI medium (Difco, BD Biosciences). Bacteria were then back-diluted 1:50 and incubated at 37°C until reaching OD_600_ ≈ 0.5–0.6. Bacteria were then washed in 1×PBS and incubated at 37°C for 15 min in 0.003% Congo red. Bacteria were added to each well to achieve MOI = 10 and centrifuged at 1000 x *g* for 10 min at room temperature to facilitate bacterial adherence. The plates were then incubated for 90 min at 37°C, 5% CO_2_. The media was removed and replaced with media supplemented with 50 μg/ml gentamicin (Quality Biological) and cells incubated at 37°C, 5% CO_2_ for 4.5 h. Cells were washed once with PBS before collecting for flow cytometry analysis.

*Salmonella* Typhimurium strain SL1344 was inoculated from a frozen stock and grown at 37°C in BHI (Difco, BD Biosciences) in a glass flask with high aeration overnight, then subcultured (1:30) and grown for 3 h at 37°C. 1 ml of bacterial suspension was then washed in PBS and resuspended in 1ml of PBS. 1:10 dilution of the bacterial suspension was used to read the optical density at 600 nm (OD600). Bacteria were added to each well to achieve MOI = 100 and incubated for 1 h at 37°C, 5% CO_2_, washed three times with PBS and incubated at 37°C, 5% CO_2_ in medium supplemented with 100 μg/ml gentamicin (Quality Biological) and cells incubated at 37°C, 5% CO_2_ for 8 h. Cells were washed again with PBS before collecting for flow cytometry analysis.

### Yellow fever virus (YFV) infection

YFV-17D-Venus infection was performed as previously described [17].

### Flow cytometry analysis

For flow cytometry analysis, cells were detached from the tissue culture plate by incubating in 150μl of Accumax Cell Dissociation Solution (Innovative Cell Technologies, Inc.) for 5 min at 37°C, transferred to V-bottom 96-well plates, pelleted by centrifugation at 800 x *g* for 5 min, resuspended in 1% paraformaldehyde (PFA) and incubated at 4°C for at least 30 min. Fixed cells were then pelleted at 800 x *g* for 5 min and resuspended in 150μl of 1×PBS containing 3% FBS. Plates were stored at 4°C if flow cytometry was not carried out immediately. Samples were analyzed using a Stratedigm S1000 flow cytometer equipped with 405nm, 488nm and 561nm lasers. Data was analyzed using FlowJo Software (Treestar).

### Measuring intracellular bacterial burden

Following *Lm* or *L*. *innocua* infection, mammalian cells were washed three times with 1×PBS and then lysed by incubating in 0.5% Triton X-100 for 5 min at room temperature, followed by vigorous pipetting to complete the lysis. Intracellular bacterial burden was determined by plating serial dilutions of suspension on BHI-agar plates, incubating at 37°C, and counting bacterial colony forming units (CFU) the next day. Additionally, serial dilutions of bacterial culture used for infection were plated to obtain the inoculated CFU. Invasion was quantified by using the following equation: [CFU recovered per well/CFU inoculated per well] x 100% = invasion and normalized to control values, if needed.

### Cell surface staining for flow cytometry

To detect surface expression of FcγRIa V450 Mouse anti-Human CD64 (BD 561202) and V450 Mouse IgG1, κ Isotype control (BD 560373) antibodies were used according to the manufacturer’s protocol. Briefly, adherent cell (4x10^5^ cells per well) were washed once with PBS, detached from the surface by incubating in 150μl of Accumax Cell Dissociation Solution (Innovative Cell Technologies, Inc.) for 5 min at 37°C, transferred to V-bottom 96-well plates, pelleted by centrifugation at 300 x *g* for 5 min, washed once PBS and staining buffer (2% FBS in 1×PBS). Cells were then resuspended in 50μl of staining buffer and 2.5μl of fluorescently tagged antibody was added. Cells were incubated for 30 min at room temperature, in the dark. After incubation, cells were washed twice in staining buffer, resuspended in 150μl of staining buffer and analyzed immediately by flow cytometry.

### Immunoblotting

Cells were washed once with PBS and lysed using RIPA Lysis and Extraction Buffer (Pierce, Thermo Fisher Scientific) supplemented with Protease Inhibitor Cocktail (Sigma). Total protein concentration was determined using the BCA Protein Assay Kit (Pierce, Thermo Fisher Scientific). Proteins were separated on SDS-PAGE and transferred to 0.45 μm nitrocellulose membranes (Biorad). Membranes were then blocked with 5% (w/v) skim milk (Difco, BD) in Tris-buffered saline with 0.1% Tween 20 (TBST) for 1 h at room temperature and immunoblotted with primary antibodies in TBST containing 5% nonfat milk at 4°C overnight, followed by incubation with appropriate secondary antibodies coupled to horseradish peroxidase (HRP) for 1 h at room temperature. Proteins were detected using ECL Western Blotting Substrate (Pierce, Thermo Fisher Scientific). The following antibodies were used in this study: anti- MYD88 (AF2928, R&D Systems), anti-E-cadherin (BD 610181, BD Biosciences), anti-c-Met (CST 4560, Cell Signaling Technology), anti-actin (a-2066, Sigma Aldrich), goat anti-rabbit (31460, Thermo Fisher Scientific), donkey anti-goat (sc-2020, Santa Cruz Biotech), goat anti-mouse (115-035-146, Jackson ImmunoResearch).

### RNA sequencing

RNA was isolated from *STAT1*-deficient fibroblasts, ectopically expressing the gene of interest, using an RNeasy Mini Kit (Qiagen) according to the manufacturer’s instructions. For each condition, two independent replicates were prepared. Further procedures were performed at the UTSW Next Generation Sequencing Core (McDermott Center). The quality of the total RNA samples was first confirmed on a 2100 Bioanalyzer (Agilent) using the total RNA 600 Nano Kit (Agilent) and amount of RNA quantified using the Qubit RNA Assay kit (Life Technologies). 4μg of total RNA with an RNA Integrity Number (RIN score) above 8, were further processed as described in TruSeq Stranded mRNA Sample Preparation Guide (Illumina). Samples were fragmented at a lower temperature than recommended (80°C for 4 min instead of 94°C for 8 min) to obtain 400-800bp libraries. Additionally, 12 PCR cycles were performed, instead of 15 cycles recommended by the protocol. Resulting libraries were analyzed on 2100 Bioanalyzer (Agilent) using DNA High Sensitivity Kit (Agilent) and quantified using Qubit. Sequencing was performed on Illumina Hiseq2500 with 100 bp paired end reads. Further procedures were performed at the UTSW Bioinformatics Core (McDermott Center). Sequencing reads were trimmed to remove adaptor sequences and low quality bases using fastq-mcf (v1.1.2–806, https://expressionanalysis.github.io/ea-utils/). Filtered reads were then mapped to human genome (hg19) using Tophat (v2.0.10) [76], guided by igenome annotations (https://ccb.jhu.edu/software/tophat/igenomes.shtml). Duplicate reads were marked but not removed. Expression abundance estimate and differential expression test were performed using Cufflinks/Cuffdiff (v2.1.1) software [76]. Differential expression was considered as statistically significant when q-value was lower than 0.05, fold change was greater than 2, and FPKM value of at least one sample was greater than 0.01. The upstream regulator analyses were generated through the use of QIAGEN’s Ingenuity Pathway Analysis (IPA, QIAGEN Redwood City, www.qiagen.com/ingenuity).

### CRISPR/Cas9-mediated *MET* and *CDH1* gene editing and clone evaluation

Guides targeting exon 3 of *CDH1* and exon 3 of *MET* were designed using the Optimized CRISPR Design Tool (http://crispr.mit.edu/), and cloned into the pX335-U6-Chimeric_BB-CBh-hSpCas9n(D10A) vector as previously described [77]. For each guide pair, 4x10^5^ HEK293A cells were seeded in a 6-well plate, and the following day were transfected with 1 μg of GFP-N3, and 1 μg of the positive and negative guides, according to the FuGENE 6 (Promega) protocol. Approximately 48 h post transfection, fluorescence-activated cell sorting (FACS) was used to deposit single GFP-positive cells into 96-well plates. Approximately 2 weeks after sorting, colonies were transferred to 24-well plates in duplicate, and screened for reduced GFP-*Lm* infection. Whole cell lysates of putatively edited clones were prepared in RIPA buffer, and western blot against either E-cadherin (BD 610181) or c-Met (CST 4560) was carried out according to a standard protocol. Further, DNA from the samples with substantially lower infection than the wild type control and no detectable E-cadherin or c-Met as determined by western blot was extracted using the Quick Extract kit. PCR using Phusion High-Fidelity DNA Polymerase (NEB) was carried out genomic primers to genotype the indels for *CDH1* and *MET* by cloning into the Zero Blunt cloning vector (Life Technologies) with subsequent Sanger sequencing at the UTSW Sequencing Core.

*CDH1* guides: 1: ATAGGCTGTCCTTTGTCGAC; 2: CTCGACACCCGATTCAAAGT

*MET* guides: 1: GTATGCTCCACAATCACTTC; 2: GGCTACACACTGGTTATCAC

### CRISPR/Cas9-mediated generation of *FCGR1A-*deficient THP-1 cells

Two guides targeting exon 3 of *FCGR1A* were designed using the Optimized CRISPR Design Tool (http://crispr.mit.edu/), and cloned into lentiCRISPR v2 vector as previously described [58]. Lentiviruses were generated as described above and used to transduce THP-1 cells. Lentivirally transduced cells were selected in 2 μg/ml puromycin for 7 days 48h after transduction. The absence of FcγRIa on the cell surface in the generated cell line was confirmed by antibody staining as described above.

*FCGR1A* guides: 1: CTGGGAGCAGCTCTACACAG; 2: CACTGTGTAGAGCTGCTCCC.

### Phagocytosis assay

*In vitro* phagocytosis assay was performed as described previously [78]. U-2 OS cells, stably expressing Fluc or FcγRIa were first transduced with lentivirus coexpressing TagRFP and Fluc, FcγRIIa or FcεRIg (γ-chain), to generate desired gene combinations. 48 h after transduction, cells were plated at 7x10^4^ cells/ml in 4-well chamber slides (Falcon). The day before the assay latex beads (3.87μm in diameter) (Bangs Laboratories, PS05N/6749) were opsonized with human IgG by washing a 10% slurry of beads in 1×PBS and mixing overnight with 1.5 mg/ml human IgG (Jackson ImmunoResearch). The day of the experiment, beads were washed in 1×PBS and labeled with an Alexa Fluor 488 AffiniPure Donkey Anti-Human IgG (H+L) (green) antibody (Jackson ImmunoResearch), while rotating at room temperature for 1h. Following secondary labeling, beads were washed, resuspended in DMEM and added to cells in chamber slides. Slides were centrifuged at 300 x *g* for 1 min, and then placed 37°C for 90 min. After the incubation, slides were placed on ice and washed with ice-cold medium to inhibit further phagocytosis. Extracellular beads were then labeled with DyLight 405 AffiniPure Donkey Anti-Human IgG (H+L) (blue) antibody (Jackson ImmunoResearch) for 10 min on ice. Cells were washed 5 times with ice-cold 1×PBS and fixed with 3.7% PFA for 20 min at room temperature. Next, cells were washed with 1×PBS and incubated with 100 mM glycine for 10 min at room temperature to quench PFA. All samples were washed twice with 1×PBS and chamber removed from the slide. When the excess liquid dried, the coverslips were mounted on the samples with ProLong Gold reagent (Molecular Probes, Life Technologies). Samples were observed with a fluorescent microscope Zeiss Observer Z1. Numbers of green and blue beads were counted for 80 Red Fluorescent Protein (TagRFP)-positive cells per sample, two technical replicates per gene. Phagocytosis efficiency was measured as a percentage of internalized beads, determined by subtracting the number of extracellular (blue) beads from the total (green) beads, divided by the number of total (green) beads.

### Agarose overlay (plaque) assay

Cells were plated at 1.4x10^5^ cells/well in a 12-well plate and transduced the next day with FcγRIa or Fluc-encoding lentiviruses as described above. Two days post-transduction, cells were infected with wild type *Lm* for 1 hr (MOI = 0.015, 0.05, 0.1), washed with medium, supplemented with 50 μg/ml gentamicin (Quality Biological), and then gently overlaid with 1.5ml/well of DMEM, containing with 10% FBS, 0.4% agarose, and 20 μg/ml gentamicin (Quality Biological). The overlay was allowed to stabilize for 15 min at room temperature, when plates were moved back to an incubator at 37°C. Foci of *Lm* infection were visualized 30 h after initial infection by adding 200μl of 5mg/ml (3-(4, 5-dimethylthiazolyl-2)-2,5-diphenyltetrazolium bromide (tetrazolium MTT) (Sigma) solution to each well and incubating at 37°C for 3 h. Plates were scanned and foci of infection quantified using ImageJ software.

### Scanning electron microscopy

Cells were plated at 1.4x10^5^ cells/well in a 12-well plate and transduced the next day with lentiviruses as described above. Two days after transduction cells were split 1:2 on circular glass coverslips in 12-well plates, and the next day infected with *Lm*, according to the standard protocol. After infection samples were fixed in 2.5% glutaraldehyde in 0.1 M cacodylate buffer for a minimum of 2 h. Further procedures were performed at the UTSW Electron Microscopy Facility. Fixed cells were rinsed in the fixation buffer and fixed with Osmium tetroxide as secondary fixative. After several water rinses they were dehydrated in serial concentrations (50%, 70%, 85%, 95%, 100%), and critical point dried. The samples were coated for 30s with gold palladium and viewed in the Zeiss Sigma VP FE scanning electron microscope. Images were acquired using the Secondary Electron 2 (SE2) detector.

### NF-κB reporter activation assay

*STAT1*-deficient fibroblasts were seeded into 48-well plates at a density of 2.5x10^4^ per well and transduced the following day with lentivirus expressing the gene of interest. 24 h later cells were transfected with 200 ng of the reporter plasmid pNF-kB–luciferase and 150 ng normalization vector pLacZ (to correct for transfection efficiency using a beta-galactosidase assay). 24 h after transfection, cells were lysed and luciferase was measured according to manufacturer protocol (Luciferase Assay System, Promega). LacZ expression was measured in a β-Galactosidase Activity Assay with ortho-Nitrophenyl-β-galactoside (ONPG), and used to normalize luciferase values for each sample.

### Statistical analysis

All experiments were performed in as three independent replicates, unless otherwise stated. For experiments where only two groups of samples were compared, unpaired t-test was used to determine if difference between groups was statistically significant. To determine statistical significance in experiments with three or more groups of samples, one-way analysis of variance (ANOVA) with Dunnett’s procedure for multiple comparisons was used. Data analysis was performed in GraphPad Prism software.

## Supporting Information

S1 Fig Expression of firefly luciferase (Fluc) in host cells does not affect *Lm* infectivity. Infectivity of *Lm* in HEK293A cells non-transduced or transduced with lentivirus co-expressing TagRFP and Fluc, and infected for 3 h following 1.5 h initial infection. Infectivity was measured by flow cytometry and presented as percentage of GFP-positive (*Lm* infected cells). Transduction level with Fluc-expressing lentivirus was >95%. Error bars represent s.d., n = 3, statistical significance was determined by t-test (n.s., not significant).
(TIF)

S2 Fig Characterization of the *CDH1/MET*-deficient HEK293A clone. (A) Western blot analysis of wild type HEK2939A, *CDH1/MET*-deficient HEK293A (clone P4E4) and Caco-2 cell lysates stained for E-cadherin. Equal amounts of each lysate (30μg total protein as measured by BCA assay) were loaded per lane. (B) Exon structure of human CDH1 gene, chromosome 7. Exon 3 was targeted for CRISPR/Cas9-mediated gene editing. (C) Sequence confirmation of the single-cell sorted clone used in Fig 6. The wild type reference sequence is shown on top, with the guide sequences underlined and Protospacer Adjacent Motif (PAM) highlighted in bold. Sequencing revealed 3 distinct alleles with frameshift insertions or deletions. (D) Western blot analysis of wild type HEK2939A and *CDH1/MET*-deficient HEK293A (clone P4E4) cell lysates stained for c-Met. Equal amounts of each lysate (30μg total protein as measured by BCA assay) were loaded per lane. (E) Exon structure of human MET gene, chromosome 16. Exon 3 was targeted for CRISPR/Cas9-mediated gene editing. (F) Sequence confirmation of the single-cell sorted clone used in Fig 6. The wild type reference sequence is shown on top, with the guide sequences underlined and PAM highlighted in bold. Sequencing revealed 4 distinct alleles with frameshift insertions or deletions. (G) Infectivity of wild type Lm in wild type (WT) and *CDH1/MET*-deficient (KO) HEK293A cells, Error bars represent s.d., n = 3
(TIF)

S3 Fig FcγRIa does not enhance infectivity of *Shigella flexneri* and *Salmonella* Typhimurium. (A) Infectivity of *Shigella flexneri* in *STAT1*-deficient fibroblasts transduced with lentivirus co-expressing TagRFP and Fluc or FcγRIa, and infected for 4.5 h following 1.5 h initial infection. Infectivity was measured as in Fig 2C, error bars represent s.d., n = 3 (n.s., not significant). (B) Infectivity of *Salmonella* Typhimurium in *STAT1*-deficient fibroblasts transduced with lentivirus co-expressing TagRFP and Fluc or FcγRIa, and infected for 8 h following 1 h initial infection. Infectivity was measured as in Fig 2C, error bars represent s.d., n = 3 (n.s., not significant).
(TIF)

S4 Fig Alignment of FcγRIa protein sequences from different species. The amino acid alignment was performed using Clustal Omega and visualized using ESPript 3.0 server http://espript.ibcp.fr [79]. Highly conserved residues are shown in red text and boxed in blue; positions that are identical between the receptors are highlighted with a red background.
(TIF)

S1 Table Related to Fig 2. Large-scale ISG screen: *Lm* infection in *STAT1*-deficient fibroblasts shown as Percent infected (percent of GFP-positive cells in RFP-positive population) cells and Z-score of infection.
(XLSX)

S2 Table Related to Fig 3. Tab 1 Differential gene expression between *STAT1*-deficient fibroblasts transduced with lentivirus expressing Fluc or MYD88. Tab 2 Upstream regulators identified by Ingenuity Pathway Analysis for MYD88-regulated genes.
(XLSX)

S3 Table Related to Fig 4. Tab 1 Differential gene expression between *STAT1*-deficient fibroblasts transduced with lentivirus expressing Fluc or TRIM14. Tab 2 Upstream regulators identified by Ingenuity Pathway Analysis for TRIM14-regulated genes.
(XLSX)

S4 Table Related to Fig 4. Tab 1 Differential gene expression between *STAT1*-deficient fibroblasts transduced with lentivirus expressing Fluc, uninfected or infected with *Lm*. Tab 2 Upstream regulators identified by Ingenuity Pathway Analysis for genes, regulated by *Lm* infection.
(XLSX)
