# Supplementary figures and images for: Cell-Based Screen Identifies Human Interferon-Stimulated Regulators of Listeria monocytogenes Infection
Source: PLoS Pathog. 2016 Dec 21;12(12):e1006102. doi: 10.1371/journal.ppat.1006102 (PMC5176324; doi:10.1371/journal.ppat.1006102)

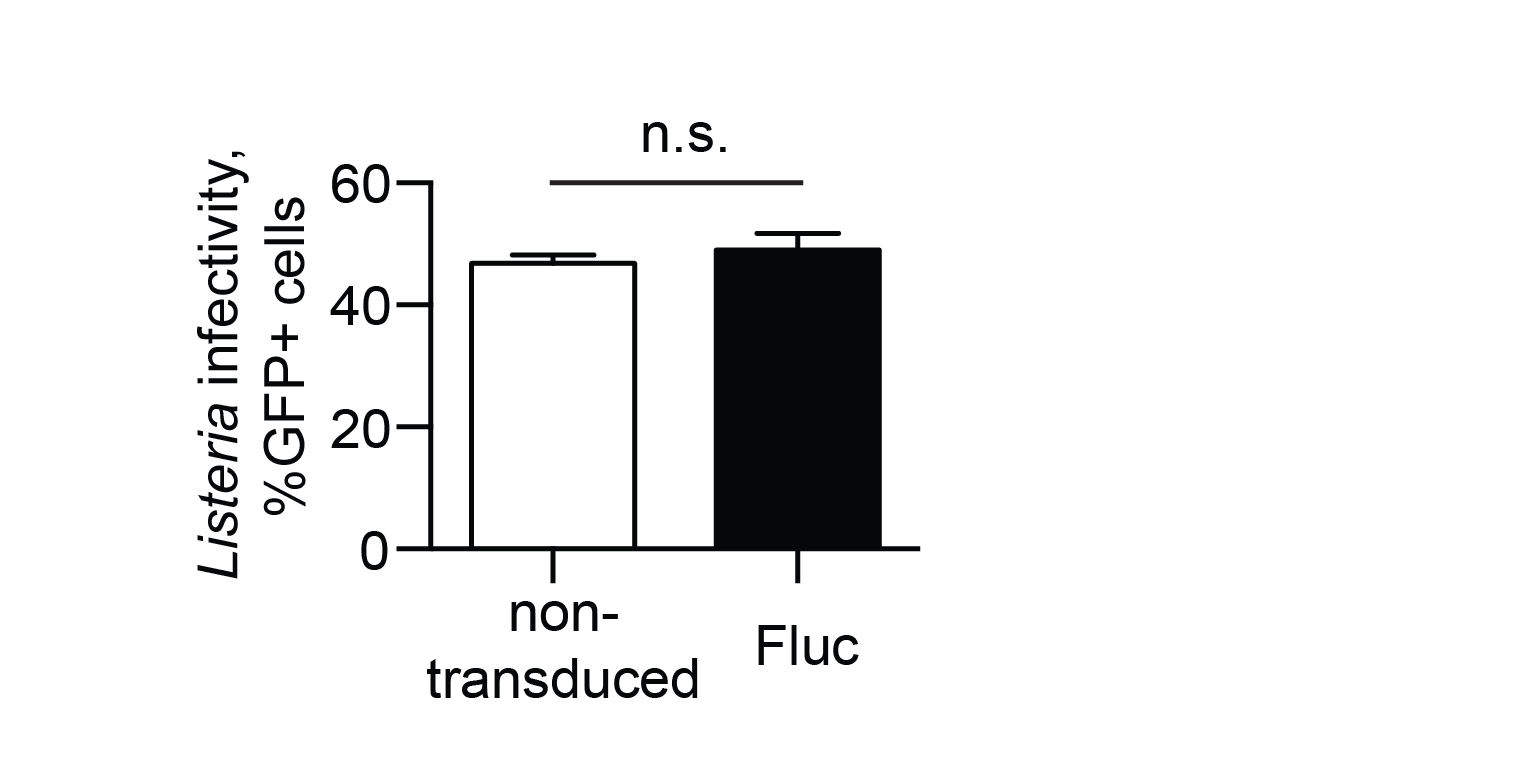

Supplement: S1 Fig — Infectivity of Lm in HEK293A cells non-transduced or transduced with lentivirus co-expressing TagRFP and Fluc, and infected for 3 h following 1.5 h initial infection. Infectivity was measured by flow cytometry and presented as percentage of GFP-positive (Lm infected cells). Transduction level with Fluc-expressing lentivirus was >95%. Error bars represent s.d., n = 3, statistical significance was determined by t-test (n.s., not significant). (TIF) [file ppat.1006102.s001.tif]

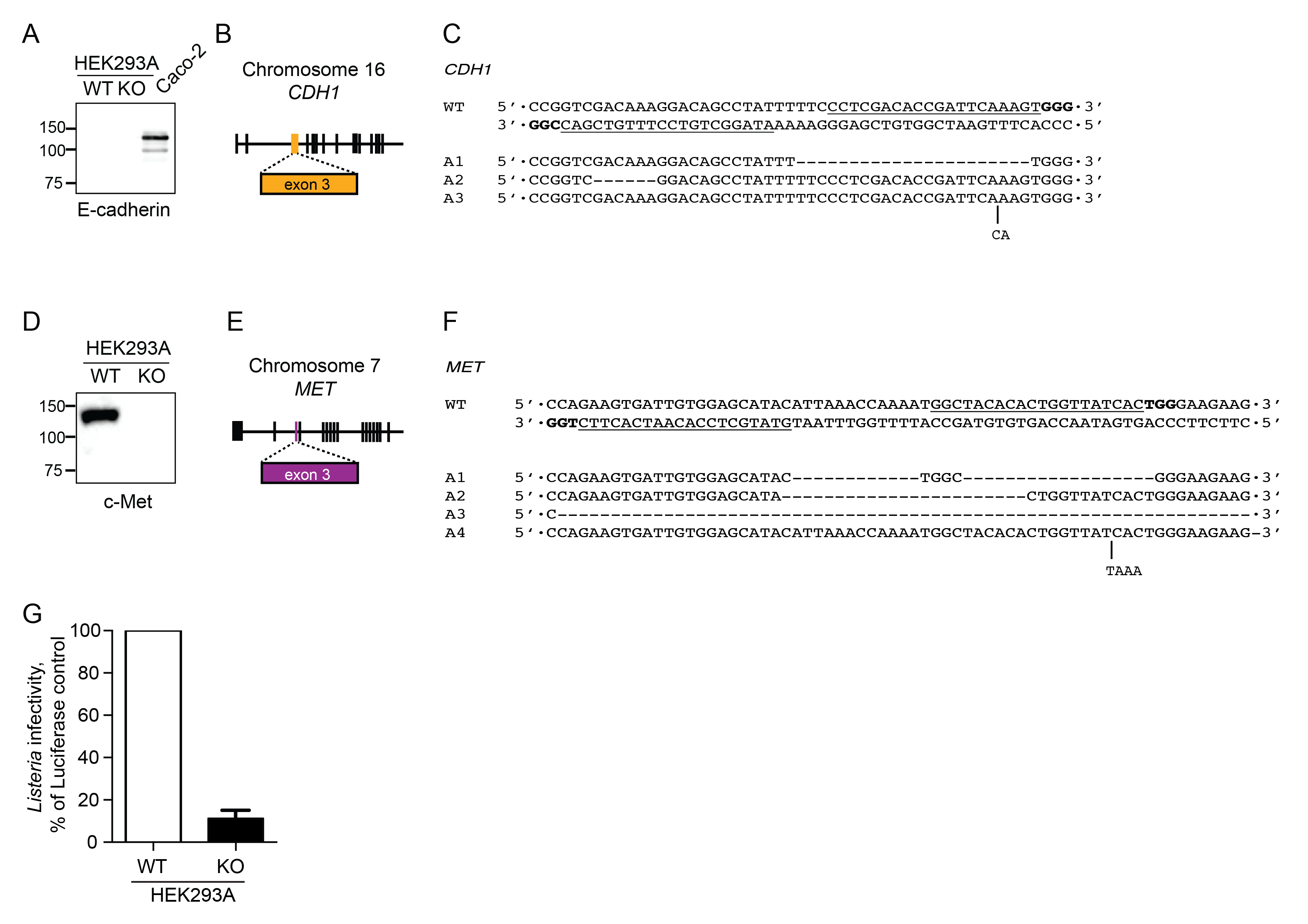

Supplement: S2 Fig — (A) Western blot analysis of wild type HEK2939A, CDH1/MET-deficient HEK293A (clone P4E4) and Caco-2 cell lysates stained for E-cadherin. Equal amounts of each lysate (30μg total protein as measured by BCA assay) were loaded per lane. (B) Exon structure of human CDH1 gene, chromosome 7. Exon 3 was targeted for CRISPR/Cas9-mediated gene editing. (C) Sequence confirmation of the single-cell sorted clone used in Fig 6. The wild type reference sequence is shown on top, with the guide sequences underlined and Protospacer Adjacent Motif (PAM) highlighted in bold. Sequencing revealed 3 distinct alleles with frameshift insertions or deletions. (D) Western blot analysis of wild type HEK2939A and CDH1/MET-deficient HEK293A (clone P4E4) cell lysates stained for c-Met. Equal amounts of each lysate (30μg total protein as measured by BCA assay) were loaded per lane. (E) Exon structure of human MET gene, chromosome 16. Exon 3 was targeted for CRISPR/Cas9-mediated gene editing. (F) Sequence confirmation of the single-cell sorted clone used in Fig 6. The wild type reference sequence is shown on top, with the guide sequences underlined and PAM highlighted in bold. Sequencing revealed 4 distinct alleles with frameshift insertions or deletions. (G) Infectivity of wild type Lm in wild type (WT) and CDH1/MET-deficient (KO) HEK293A cells, Error bars represent s.d., n = 3 (TIF) [file ppat.1006102.s002.tif]

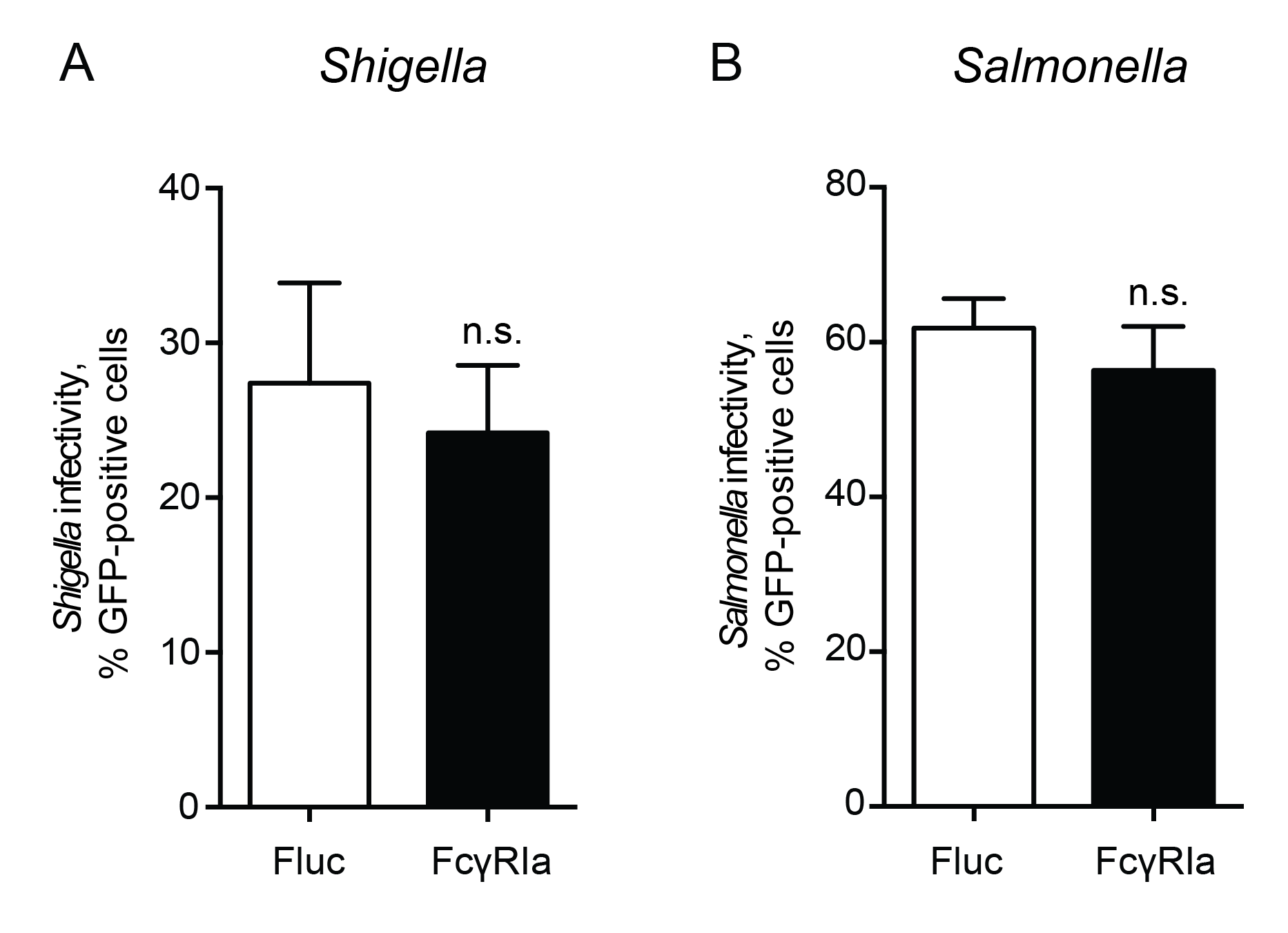

Supplement: S3 Fig — (A) Infectivity of Shigella flexneri in STAT1-deficient fibroblasts transduced with lentivirus co-expressing TagRFP and Fluc or FcγRIa, and infected for 4.5 h following 1.5 h initial infection. Infectivity was measured as in Fig 2C, error bars represent s.d., n = 3 (n.s., not significant). (B) Infectivity of Salmonella Typhimurium in STAT1-deficient fibroblasts transduced with lentivirus co-expressing TagRFP and Fluc or FcγRIa, and infected for 8 h following 1 h initial infection. Infectivity was measured as in Fig 2C, error bars represent s.d., n = 3 (n.s., not significant). (TIF) [file ppat.1006102.s003.tif]

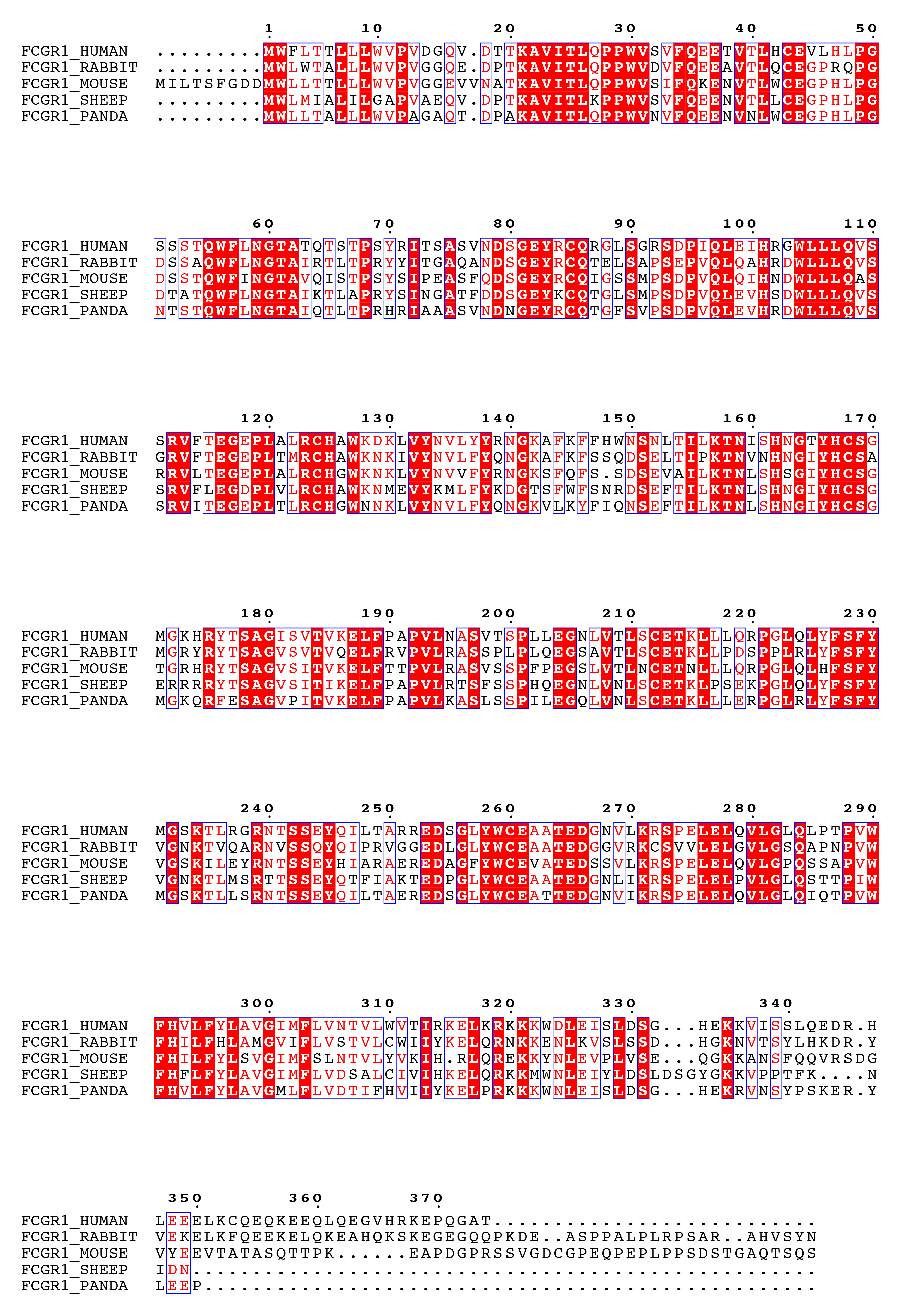

Supplement: S4 Fig — The amino acid alignment was performed using Clustal Omega and visualized using ESPript 3.0 server http://espript.ibcp.fr [79]. Highly conserved residues are shown in red text and boxed in blue; positions that are identical between the receptors are highlighted with a red background. (TIF) [file ppat.1006102.s004.tif]
